# Functional traits explain growth response to successive hotter droughts across a wide set of common and future tree species in Europe

**DOI:** 10.1111/plb.70024

**Published:** 2025-05-07

**Authors:** L. Kretz, F. Schnabel, R. Richter, A. Raabgrund, J. Kattge, K. Andraczek, A. Kahl, T. Künne, C. Wirth

**Affiliations:** ^1^ Systematic Botany and Functional Biodiversity, Life Sciences Leipzig University Leipzig Germany; ^2^ Department Conservation Biology and Social‐Ecological Systems Helmholtz Centre for Environmental Leipzig Germany; ^3^ Chair of Silviculture, Faculty of Environment and Natural Resources University of Freiburg Freiburg Germany; ^4^ German Centre for Integrative Biodiversity Research (iDiv) Halle‐Jena‐Leipzig Leipzig Germany; ^5^ Geoinformatics and Remote Sensing, Institute for Geography Leipzig University Leipzig Germany; ^6^ Max Planck Institute for Biogeochemistry Jena Germany

**Keywords:** ARBOfun, climate change, drought tolerance, forest, shoot increment

## Abstract

- In many regions worldwide, forests increasingly suffer from droughts. The ‘hotter drought’ in Europe in 2018, and the consecutive drought years 2019 and 2020 caused large‐scale growth declines and forest dieback. We investigated whether tree growth responses to the 2018–2020 drought can be explained by tree functional traits related to drought tolerance, growth and resource acquisition.
- We assessed the growth response, that is, growth during drought compared to pre‐drought conditions of 71 planted tree species, using branch shoot increments. We used gap‐filled trait data related to drought tolerance (P50, stomatal density, conductivity), resource acquisition (SLA, LNC, C:N, A_max_) and wood density from the TRY database to explain growth responses, while accounting for differences in growth programmes (spring vs. full‐season growing species).
- We found significantly reduced growth during the 2018 drought across all species. Legacy effects further reduced growth in 2019 and 2020. Gymnosperms showed decreasing growth with increasing P50 and acquisitiveness, such as high SLA, LNC, and A_max_. Similar results were found for angiosperms, however, with a less clear pattern. Four distinct response types emerged: ‘Sufferer’, ‘Late sufferer’, ‘Recoverer’ and ‘Resister’, with gymnosperms predominately appearing as ‘Sufferer’ and ‘Late sufferer’. ‘Late sufferers’ tended to be *spring growing* species.
- This study provides evidence for significant growth reductions and legacy effects in response to consecutive hotter droughts, which can be explained by functional traits across a wide range of tree species when accounting for fundamental growth programmes. We conclude that high drought tolerance bolsters growth reductions, while acquisitive species suffer more from drought.

## Graphical abstract

Functional traits can explain significant tree growth reductions in response to the 2018–2020 drought for a wide set of 71 species including angiosperms and gymnosperms. Moreover, four distinct response types emerged: ‘Sufferer’, ‘Late sufferer’, ‘Recoverer’ and ‘Resister’, with gymnosperms predominately appearing as ‘Sufferer’ and ‘Late sufferer’.

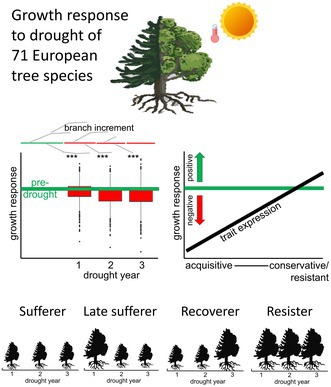

## INTRODUCTION

Forest productivity is declining in many regions of the world, as trees suffer from more intense and frequent drought events caused by climate change (Allen *et al*. 2010; IPCC 2014; McDowell *et al*. 2020). The negative impacts on forests are particularly pronounced during so‐called ‘hotter droughts’, compound events characterised by low precipitation and simultaneous heatwaves (Allen *et al*. 2015). Hotter droughts increase soil water depletion and canopy temperature, reducing plant turgor pressure (Salomón *et al*. 2022; Knutzen *et al*. 2025). As a result, physiological processes are compromised, leading to growth declines (Allen *et al*. 2015; Salomón *et al*. 2022). Such growth declines often precede large‐scale tree mortality events, which are ultimately exacerbated by climate change‐induced insect and pathogen outbreaks (Allen *et al*. 2015; McDowell *et al*. 2020). Thus, hotter droughts negatively affect many ecosystem functions and services of forests, such as carbon sequestration (Buras *et al*. 2020; Senf *et al*. 2020) and transpirative cooling (Richter *et al*. 2021), and may induce strong changes in species composition (Schuldt *et al*. 2020). However, we have only a limited mechanistic understanding of how intense drought events cause growth reductions across a wide range of tree species. Furthermore, we know little about which functional properties of tree species help to develop trait‐based models to generalise responses to hotter droughts across broad taxonomic gradients and to parametrize models predicting growth response and eventual mortality risk (Adams *et al*. 2017).

In 2018, Central Europe experienced a hotter drought, the most extreme in Europe since the beginning of climate records (Schuldt *et al*. 2020; Zscheischler & Fischer 2020). The hotter drought conditions continued into 2019 and even into 2020 in many Central European regions (Rakovec *et al*. 2022). These three consecutive drought years (hereafter referred to as the ‘2018–2020 drought’) may mark the beginning of a new era of compound climate extremes, consistent with climate change models that predict hotter, drier and more extreme climate conditions, especially in the summer months, for Central Europe in the 21st century (Reichstein *et al*. 2013; IPCC 2014; Trenberth *et al*. 2014; Zscheischler & Seneviratne 2017; Samaniego *et al*. 2018). Consecutive and hotter droughts induce prolonged stress, especially when interacting with outbreaks of fungal pathogens and insects (Hari *et al*. 2020; Kleine *et al*. 2021; Schnabel *et al*. 2022; Thonfeld *et al*. 2022).

Here we aim to understand tree growth responses to the 2018–2020 drought. We focus on potential growth responses during the second and third drought years (2019 and 2020), as in those years, the effects of consecutive droughts overlap with the legacy effects of the initial drought in 2018. Legacy effects of the 2018 drought, such as associated damage to the tree water transport system (Anderegg *et al*. 2013), may limit the ability of trees to cope with and recover from subsequent drought years. Such drought legacy effects may still negatively affect trees even 5 years after the actual drought event (Anderegg *et al*. 2015; Kannenberg *et al*. 2018; Zweifel & Sterck 2018; Schnabel *et al*. 2022; Sangüesa‐Barreda *et al*. 2023). Therefore, lower growth can be expected in the consecutive drought years 2019 and 2020.

Under drought conditions, plants face a trade‐off between carbon gain and water loss (Cowan & Farquhar 1977). Key drought‐tolerance traits that influence this trade‐off are related to species' cavitation resistance and stomatal control, which drive tree responses, including growth reduction and, ultimately, mortality under drought (McDowell *et al*. 2008; Adams *et al*. 2017; Schuldt *et al*. 2020; McDowell *et al*. 2022). Indeed, previous studies have shown that drought‐tolerance traits, such as P50 (the pressure at which 50% of the hydraulic system conductivity is lost due to cavitation) (Adams *et al*. 2017; Guillemot *et al*. 2022), and stomatal control traits, such as stomatal conductance, can help in understanding growth declines during drought (Schnabel *et al*. 2021, 2022). Specifically, tree species with functional traits related to high cavitation resistance, such as a low P50 (Jarbeau *et al*. 1995; Choat *et al*. 2018), may have a lower growth reductions during drought. Similarly, stomatal control traits that capture a gradient, ranging from species that save water and close their stomata early, to species that continue to spend water and keep their stomata open despite of drought (McDowell *et al*. 2008; Klein 2014), may influence growth responses to drought. We assume that species with small stomata size, high stomata density and lower mean stomatal conductance are associated with faster and more precise stomatal control (water‐saving species), which should result in enhanced drought adaptation and comparatively low growth reductions during droughts (Martin‐StPaul *et al*. 2017; Wankmüller & Carminati 2022). Furthermore, traits related to growth and resource acquisition, such as the Leaf Economics Spectrum (LES), representing the slow–fast gradient of plant growth (Reich 2014; Guillemot *et al*. 2022), may explain growth responses during drought. In particular, traits related to conservative resource use and slow growth, such as a high carbon to nitrogen ratio (C:N), may indicate smaller growth reduction under drought (Wright *et al*. 2004; Reich 2014; Choat *et al*. 2015). In contrast, LES traits related to acquisitive resource use and rapid growth, such as high specific leaf area (SLA), may experience larger growth reductions during drought (Wright *et al*. 2004; Reich 2014; Díaz *et al*. 2016). Besides LES traits, wood density includes different wood properties and is associated with mechanical strength and stem water transport (Chave *et al*. 2009; Zanne *et al*. 2010). Thus high wood density may indicate lower growth reduction under drought (Serra‐Maluquer *et al*. 2022), although contrasting responses have also been observed (Hoffmann *et al*. 2011).

To guide management decisions and to improve the predictive capacity of forest models, it is important to understand the response of Central European tree species to the 2018–2020 drought. Moreover, this is true not only for the dominant tree species of today's forests, but also for the many subordinate or biogeographically neighbouring tree species that may form the forest under future climate regimes. So far, establishing relationships between the functional properties of tree species and their responses to novel climates is challenging. Existing studies investigating the trait‐based mechanisms underpinning drought effects on tree growth (Bose *et al*. 2020; Liu *et al*. 2022; Schnabel *et al*. 2022) have typically been conducted on relatively few tree species and at varying spatial scales. Furthermore, the national forest inventories are of limited use for two reasons: (i) they do not provide the necessary temporal resolution required to capture the sequence of growth responses to drought, and (ii) the Central European managed forest landscape is dominated by a few commercially important tree species, such as Norway spruce (*Picea abies*), Scots pine (*Pinus sylvestris*), European beech (*Fagus sylvatica*) and pedunculate oak (*Quercus robur*), which account for 73.5% of the forest area (BWI, 2012). In contrast, rare species with greater potential for forestry in drier and hotter climates, such as the chequer tree (*Sorbus torminalis*) and downy oak (*Quercus pubescens*) are barely recorded (Kunz *et al*. 2018; Buras & Menzel 2019). Therefore, studying a wide range of species growing under comparable conditions is of crucial importance for improving our understanding of tree growth responses to drought.

Here, we investigate the effects of the three consecutive drought years, 2018–2020, on the growth of a large set of 71 tree species (Table S16) planted in the ARBOfun research arboretum. ARBOfun was designed to study responses to climate variability for a large number of tree species, while controlling for the confounding effects of environmental variation. The experiment includes gymnosperm and angiosperm tree species, covering a wide range of native and introduced Central European species. Each species is replicated five times in a wide stand without competition and grown in similar soil conditions. We aim to provide new insights into the growth response to drought of an unprecedented set of tree species and to test whether the strength and type of growth responses can be predicted by tree functional traits related to drought tolerance and resource acquisition capacity. We quantify growth from a morphological reconstruction of shoot increment using bud scar analysis (Girard *et al*. 2012; Camarero *et al*. 2015; Liu *et al*. 2022). When using this method it is important to control for differences in growth programmes (Thomas 2000; Barthélémy & Caraglio 2007). In some species the following year's shoot is pre‐formed and shoot growth can only respond to the climate by changing cell size within the shoot tissues. These species usually complete their growth within 1 to 2 months in spring/early summer (Marks 1975; Brown & Sommer 1992). Other species continue to grow through summer and autumn or have lammas shoots. For these species new leaves and internodes are added during the growing season, depending on prevailing abiotic conditions, allowing for a stronger climate effect on shoot growth. Here, we accounted for these differing growth programmes, which were identified based on weekly observations of leaf production, allowing us to classify tree species in “*spring growing*” or “*full‐season growing*” species.

We hypothesized that:

1. The 2018–2020 drought reduced tree growth, with a larger growth reduction in 2019 and 2020 compared to 2018, due to legacy effects.
2. Tree species with *full‐season growth* show a bigger reduction in growth and have higher sensitivity of their growth response to functional traits than *spring growing* species. This difference is most pronounced in 2018.
3. Tree species with functional traits indicating high drought tolerance show less growth reduction under drought than drought‐intolerant species.
4. Tree species with resource acquisition traits favouring rapid growth are more susceptible to drought and show larger growth reduction during drought than tree species with traits indicating conservative resource use.

## MATERIAL AND METHODS

### Experimental design and study site

The ARBOfun research arboretum is located south of Leipzig (Saxony, Germany, 51°16′ N, 12°30′ E). The experiment was established in 2012 on 2.5 ha of former extensively used arable land, with the soil type being a Luvisol. A set of 69 species was planted in 2012, and 31 additional species were added in 2014, resulting in a total of 100 tree species. Each species is randomly replicated five times within a block design, with each block containing one individual per species (Fig. S2).

Tree individuals are spaced 5.8 m apart in a checkerboard pattern to avoid competition in the early years of the experiment. Due to mortality, mostly unrelated to drought (e.g. vole damage to roots), not all species had five available replicates. The meadow between the trees is mown twice a year. The tree species selected represent the diversity of woody species native to Europe, with a gradient from hemi‐boreal to sub‐Mediterranean forests. ARBOfun also includes selected species from North America and Asia, which are often planted in plantations or cities. Like the sub‐Mediterranean species, many of these are considered relevant as future tree species under climate change in Central Europe (Table S16).

**Fig. 1:**
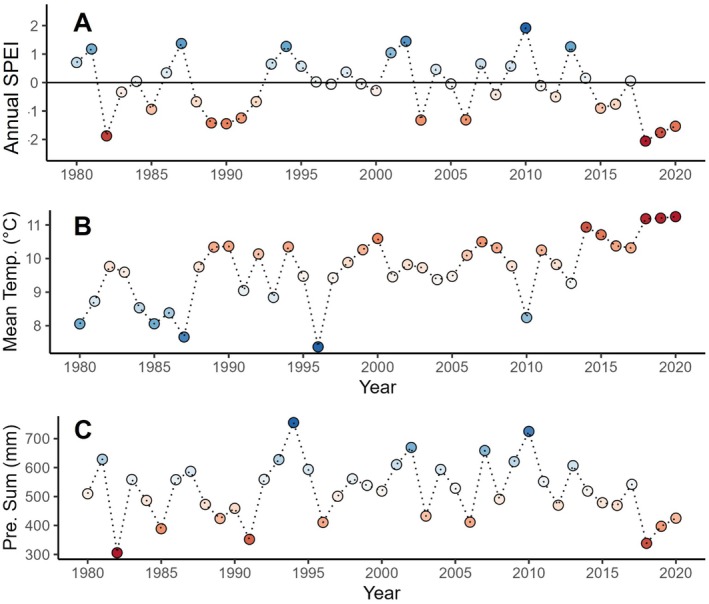
(A) Annual Standardised Precipitation–Evapotranspiration Index (SPEI), (B) mean annual temperature and (C) annual precipitation sum. The annual SPEI (SPEI12) is calculated for the whole year, from January to December. The zero line is the reference period 1981–2010. Blue dots indicate positive SPEI values, while red dots show negative SPEI values. The mean temperature and precipitation sum are calculated over the whole year. Also, here, blue and red indicate higher and lower values compared to the reference period, respectively.

The study site is at an altitude of 150 m a.s.l. in the transition zone from maritime to continental climate. Mean annual precipitation at the site is ca. 520 mm, and mean annual temperature is 9.7°C (1980–2020; DWD Climate Data Center [CDC], Station Leipzig/Halle, ID 2932). To characterise climatic conditions at our study site (Fig. 1), we used climate data derived from the aforementioned weather station, which is closest to the experiment (about 30 km northwest) and has complete temporal records. We examined monthly temperature and precipitation, as well as the standardised water balance of precipitation minus potential evapotranspiration using the Standardised Precipitation Evapotranspiration Index (SPEI; Vicente‐Serrano, et al. 2010). The SPEI is a widely used drought index (Hari *et al*. 2020; Schwarz et al., 2020) that quantifies drought severity according to the intensity and duration of a drought across time (Vicente‐Serrano et al., 2010). We computed three different SPEI lengths using the SPEI package (Beguería & Vicente‐Serrano, 2011) in R: SPEI3, which captures the water balance during the main vegetation period (May–July), SPEI6 during the whole vegetation period (April–September) and SPEI12 throughout the whole year (January–December). Potential evapotranspiration was calculated with the FAO‐56 Penman‐Monteith equation (Beguería & Vicente‐Serrano, 2011) using the following DWD data: monthly means of daily minimum temperature, daily maximum temperature, wind speed, cloud cover, atmospheric surface pressure, relative humidity, vapour pressure, and station altitude and latitude.

### Tree sampling and trait measurements

For the present study, we measured shoot increment for 71 tree species (Table S16). Measurements were made in spring 2021. We used the scars of bud scales to retrospectively measure shoot increment of three lateral branches per tree from the year 2020 back to the year 2016 (Fig. S3). To measure lateral branches, we first selected and measured the longest lateral branch that was south‐facing and at approximately ¼ of the total tree height. In addition, a second lateral branch was selected counter‐clockwise around the tree at an angle of 120° from the first branch, while a third lateral branch was selected in the same way starting from the second branch. Multiple branch flushes per year were summed. For the present study, we only included species with at least two replicate trees with at least two branch measurements that could be dated back to at least 2017. This resulted in a total of 850 measured branches on 284 tree individuals.

The tree growth response to drought is calculated for the drought years 2018, 2019, or 2020 compared to the pre‐drought growth, which we calculated as mean growth in the reference years 2016 and 2017 (growth for 2016 was only available for 81% of the branches). The years 2016 and 2017 were neither exceptionally wet nor dry, nor preceded by severe drought years which could have caused legacy effects (Fig. 1; Fig S4). The growth response index took the following form:

$${\mathrm{GR}}_{\mathrm{dr}.y}=\frac{b_{\mathrm{dr}.y}}{b_{\mathrm{pre}.\mathrm{dr}.y}}$$

Where, GR is the growth response, and *b* is the branch increment length, while *dr.y* is the drought year, and *pre.dr.y* is the pre‐drought reference period. Note, that we used the log‐transformed GR_*dr.y*_ values in all analyses to fulfil model assumptions (see below) and that values of GR_*dr.y*_ > 0 thus indicates higher growth relative to pre‐drought growth, while values of GR_*dr.y*_ < 0 indicate lower growth relative to pre‐drought growth.

As we expect that tree species with different growth programmes have different predispositions in their response to climate conditions (Marks 1975; see hypothesis 2), we collected phenological data so as to distinguish between *spring growing* and *full‐season growing* species. As part of a phenological survey, three individuals of each species in ARBOfun were inspected each week during 2024, and signs of active growth, that is, visibility of not fully expanded leaves, needles and shoots recorded. For 10 species, only two individuals were available, and 2 weeks of observations were missing for the entire survey. We classified the growth programme into two groups: *spring growing* species (species that stop growth before midsummer [June 21st], with a maximum of one observation of active growth in later weeks to account for potential observer error), and *full‐season growing* species (species that continue growing after midsummer, with at least two observations of active regrowth). We classified each species accordingly to the median of individuals per species (all species with only two replicates had an identical growth programme). We did not use the terminology “preformed/neoformed” as clear assignment of this definition would require additional anatomical validation, that is, a comparison of the number of bud scars in the last year's shoots with the number of embryonic leaves in the terminal bud (described by Moore 1909).

We selected eight different functional traits that we expected to be key traits for predicting growth response to drought, based on the literature (Anderegg *et al*. 2019) and on their availability in the TRY database for a large number of species (Kattge *et al*. 2020): (1) P50, (2) stomatal density, (3) stomatal conductance, (4) specific leaf area (SLA), (5) leaf nitrogen content (LNC), (6) leaf carbon to nitrogen ratio (C:N), (7) maximum photosynthesis rate (A_max_), and (8) wood density.

1. P50 (MPa) describes xylem pressure at which 50% of conductivity of the hydraulic system has been lost. If xylem pressure falls below this value, the plant is at high risk of lethal embolism (Sperry & Tyree 1988; Brodribb & Cochard 2009).
2. Stomatal density (mm^−2^) is number of stomata per leaf area and can be linked to stomata size and distribution, but is also indicative of stomatal control and conductance (Klein 2014).
3. Mean stomatal conductance (mmol m^−2^ s^−1^) is conductance for water vapour per leaf area per time for stomata, and can be related to tree hydraulics and leaf water potential, but also to photosynthesis rate and, thus, mechanistically to acquisition strategy (Garcia‐Forner *et al*. 2016).
4. Specific leaf area (SLA; mm^2^ mg^−1^) is the leaf area per invested leaf biomass. It has been suggested to be negatively related to plant performance under drought (Poorter *et al*. 2009). Furthermore, it is a key trait representing high resource acquisition capacity and rapid growth in the Leaf Economics Spectrum (Wright *et al*. 2004; Reich 2014).
5. Leaf nitrogen content (LNC; mg g^−1^) is a major component of photosynthetic compounds, such as Rubisco, and thus directly affects photosynthetic capacity of leaves (Evans 1989; Reich *et al*. 1995). LNC is also one of the traits representing high resource acquisition capacity and rapid growth in the LES (Wright *et al*. 2004; Reich 2014).
6. The leaf carbon to nitrogen ratio (C:N) is, among other functions, linked to growth, but also to stress responses (Hessen *et al*. 2004). A high C:N indicates low N concentration and thus slow growth, as mentioned above, but also a high C concentration, which indicates thicker cell walls, making the species more resistant to drought stress (Wright *et al*. 2004; Reich 2014).
7. Light‐saturated maximum photosynthesis rate (A_max_; μmol g^−1^ s^−1^) is the maximum rate of carbon assimilation under non‐limited water conditions, an index of photosynthetic capacity (Anderegg *et al*. 2018; Zhu *et al*. 2018), and associated with high resource acquisition capacity (Lambers & Poorter 2004).
8. Wood density (g cm^−3^) combines various wood properties, such as mechanical strength, water storage and transport (Chave *et al*. 2009; Zanne *et al*. 2010).

### Statistical analysis

All statistical analyses were performed using the statistical software R v. 4.2.2 (R Core Team 2020). We used linear mixed‐effects models (lme function in nlme package; Pinheiro *et al*. 2023) to predict growth responses to drought across tree species. We used drought year (2018, 2019 and 2020) coded as a factor, as a fixed effect. We log‐transformed the tree growth response index to fulfil model assumptions (normality and homogeneity of variance), and used branch ID nested within tree ID as a random effect structure. We accounted for temporal autocorrelation between repeated measurements with a first‐order autocorrelation structure (corAR1), with year as a covariate, and used a post‐hoc test for comparisons between years (emmeans function in the emmeans R package, with pairwise Tukey adjustments to account for multiple comparisons; Lenth 2023). We also ran linear mixed‐effects models in the same way for gymnosperms and angiosperms separately and for each individual species. The full model is shown in equation S1a. The differences between growth programmes were accounted for by running the same linear mixed‐effects models as before but additionally including the interaction between growth programme (two‐level factor) and year (equation S1b).

We used available trait data from TRY, the Plant Trait Database (Kattge *et al*. 2020; for TRY data citation, see below). As the available data did not provide a complete trait dataset, we performed a gap‐filling to predict trait values for those traits and species that were not available. For the gap‐filling, we used a hierarchical Bayesian implementation of probabilistic matrix factorisation (BHPMF; Schrodt *et al*. 2015), which is a machine learning approach that predicts missing trait values based on the taxonomic hierarchy of the species. We used all available species data in TRY for the gap‐filling and only traits with at least 1000 available entries in the database. As a first step, we cleaned the available data on TRY, excluding non‐vascular species, juveniles, and unhealthy plants. We also excluded (i) outlier values with a distance of >5 SD from the taxonomic or functional group means (Kattge *et al*. 2011, 2020), and (ii) A_max_ and stomatal conductance values measured under conditions of non‐ambient CO_2_ (300–450 ppm), unsaturated light conditions (< 800 μmol m^−2^ s^−1^), and temperatures outside 20°C and 30°C. The data were z‐transformed and gap‐filling was performed using the BHPMF approach. In a post‐processing step, we back‐transformed the data and excluded data from the 25% quantile with the highest SD per prediction of a trait record (Fazayeli *et al*. 2014), and excluded data with a distance of >3 SD from the taxonomic and functional group means.

With the gap‐filled data, we ran linear mixed‐effects models to predict the growth response of each individual trait in interaction with drought year and growth programme. We used the same model specification and R package as used above, except for adding the respective trait (see equation S1c). We tested for an interaction between each trait and growth programme using ANOVA (R Core Team 2020). We also performed principal components analyses (PCA) on the trait data. For the PCAs, we were able to include 58 species with full trait coverage: 19 gymnosperms and 39 angiosperms. The PCAs were performed using the prcomp function (R Core Team 2020), as for the individual traits, we also used the loadings of PCA axes 1 and 2 as predictors of growth response. We used Fisher's exact test for contingency table data to analyse different response types within the clades. We used MANOVA (Multivariate Analysis of Variance) with the function manova (R Core Team 2020) to compare the response types and growth programmes within the trait spaces per clade.

## RESULTS

### Climate

We observed consecutive hotter drought conditions from 2018 to 2020, with a slight decrease in climate drought severity from 2018 to 2019 to 2020 (annual SPEI (SPEI12) values of −2.06, −1.76, −1.53, respectively; Fig. 1A). All 3 years were among the driest in the last 40 years, when considering the peak vegetation period (May–July), the full vegetation period (April–September), and the whole year (Figs. 1A, S4). The 3 drought years differed in terms of drought onset and duration, but in all e years, exceptional dry conditions (SPEI <−1; McKee *et al*. 1993) started early in the season and lasted over the season (onset in April in 2018, in February in 2019 and in March in 2020; Fig. S6). In particular, the coincidence of high temperatures (Fig. 1B) and low precipitation (Fig. 1C), as well as the consecutive nature of these droughts with never enough precipitation during 2018–2020 to refill water reservoirs (UFZ, Drought Monitor/Helmholtz Centre for Environmental Research), marked the 2018–2020 drought as exceptional.

### Growth response of all species

**Fig. 2:**
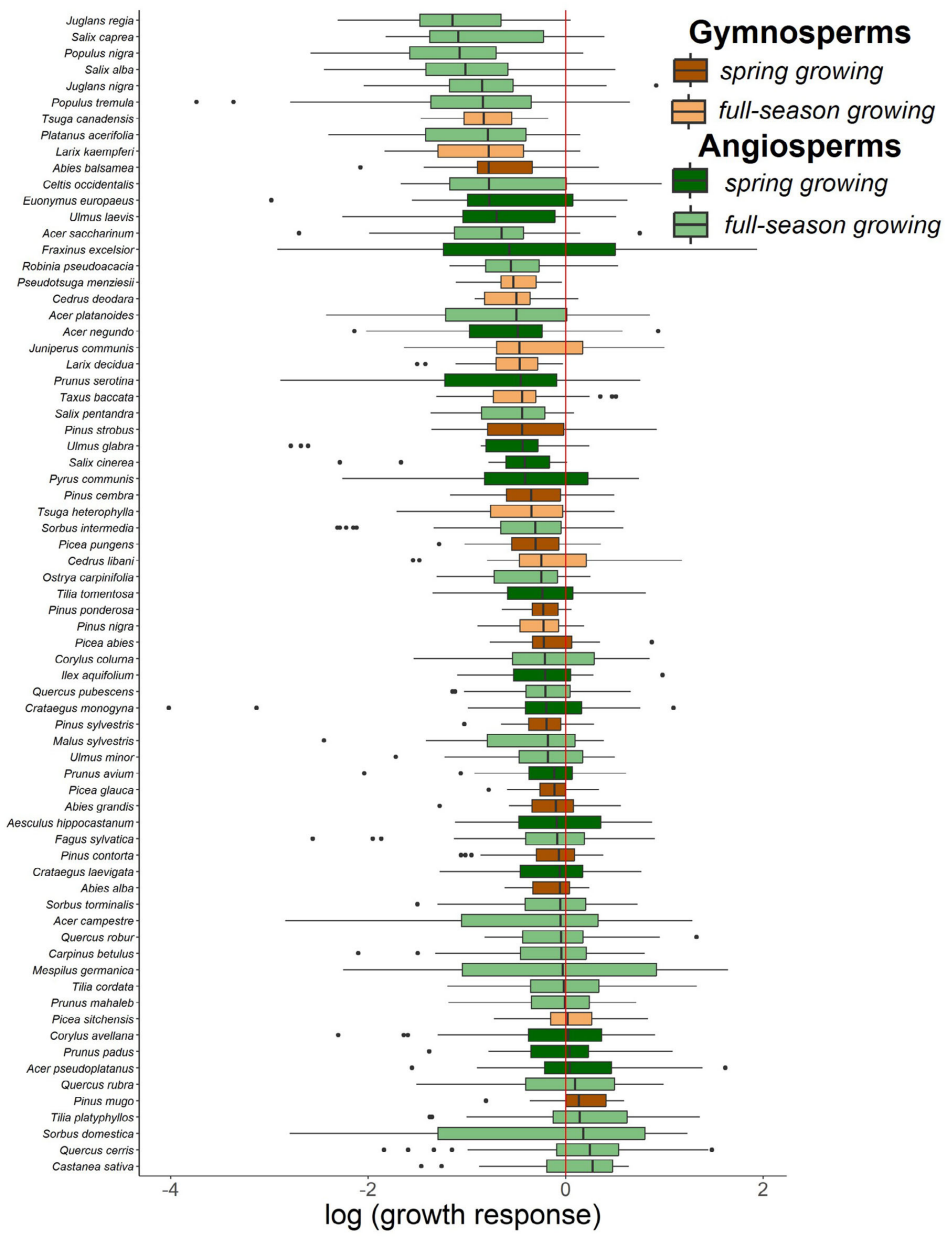
Growth response of all tree species over the drought years 2018–2020, sorted by median growth response per species. A growth response <0 (red vertical line) indicates lower growth compared to pre‐drought conditions, while a growth response >0 indicates higher growth compared to pre‐drought conditions.

The strongest growth reduction, as indicated by the most negative growth response, was measured for *Crataegus monogyna* in 2020 (log growth response −4.017), while the strongest growth increase was for *Fraxinus excelsior* also in 2020 (log growth response 1.940; Fig. 2). The median log growth response per species varied from −1.146 (*Juglans regia*) to 0.273 (*Castanea sativa*; Fig. 2), indicating reduced and increased growth compared to growth in the reference period (log growth response of 0, respectively; Fig. 2). The gymnosperm with the lowest median log growth response of −0.830 was *Tsuga canadensis* and with the highest median log growth response of 0.134 was *Pinus mugo*.

**Fig. 3:**
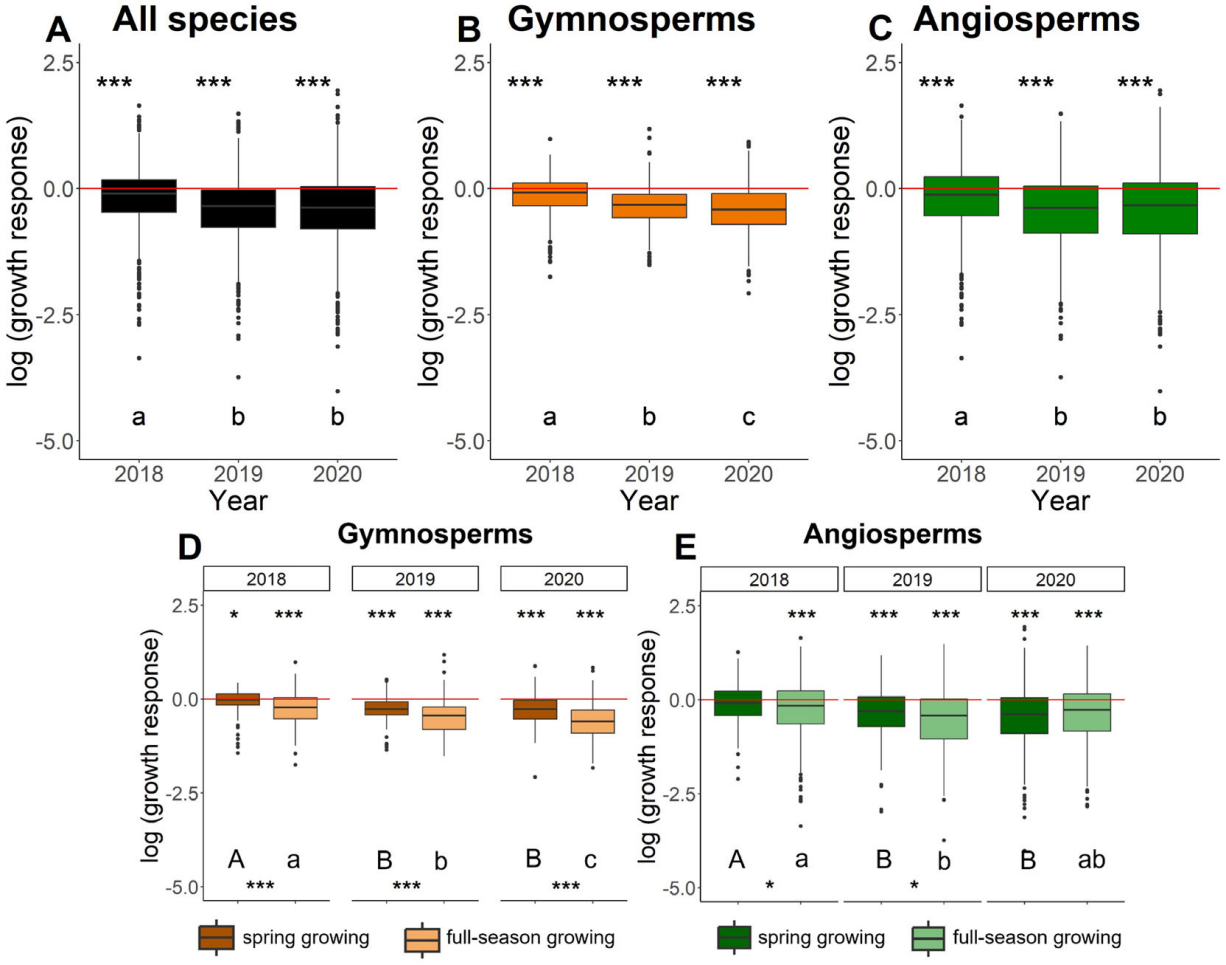
Growth response to drought. Boxplots show the growth response of tree species during the 2018–2020 drought compared to growth in the reference years (mean of 2016 and 2017), shown as a red zero line. Across species (A), asterisks indicate significant differences in growth during drought years (*p < 0.05, ***p < 0.001) compared to growth in reference years. Significant differences between years (A) were tested with a post‐hoc test and are indicated by the characters (a, b, c). (B, C) Similarly, significant reductions in growth were found when analysing gymnosperms and angiosperms separated. (D, E) Asterisks above graphs indicate significant differences in growth within the drought years and growth programme compared to growth in the reference period. Letters below the graph indicate significant differences within the growth programme between the years. Asterisks below the graphs indicate significant differences in growth between *spring growing* and *full‐season growing* species within a clade and a year.

We found significantly lower growth for all species in the years 2018 (*p* < 0.001), 2019 (*p* < 0.001) and 2020 (*p* < 0.001) compared to mean growth response in the reference years 2016 and 2017 (Fig. 3A). The interannual comparison showed that growth in 2019 (*p* < 0.001) and 2020 (*p* < 0.001) was significantly lower than in 2018, while the growth response in 2020 was not significantly different from that of 2019. The same was true when we analysed gymnosperm and angiosperm clades separately, but for the gymnosperms the interannual comparison also showed significant lower growth in 2020 compared to 2019 (*p* = 0.031; Fig. 3B,C). Across both clades and drought years, the *full‐season growing* species showed larger growth reductions than *spring growing* species, with the growth reduction being more pronounced in gymnosperms (Fig. 3D,E). The *spring growing* angiosperms showed similar growth compared to the reference period in 2018 (Fig. 3D), while *full‐season* growing angiosperms already showed a significant growth reduction (Fig. 3E). Furthermore, *full‐season growing* angiosperms did not differ significantly in their growth response in 2020 compared to 2018 (Fig. 3E), suggesting the onset of recovery.

### Response types

With the individual species models, there were recurring patterns that allowed us to classify the species *a posteriori* into four main response classes: ‘Sufferer’, ‘Late sufferer’, 'Recoverer' and ‘Resister’, based on their drought responses over the three consecutive drought years (Figs. S9, S10). The models explained 2%–77% of the variation in growth responses through their fixed effects (R^2^m) and 3%–81% through their fixed and random effects (R^2^c; Table S16).

‘Sufferer’ are species with significantly reduced growth in all drought years, 2018, 2019, and 2020, indicated by a significantly negative growth response. ‘Late sufferer’ are species that did not have significantly reduced growth in 2018, but had significantly reduced growth in 2020 at the latest. The ‘Recoverer’ are species with significantly reduced growth in 2018 and/or 2019, but no significantly reduced growth in 2020 (Fig. S10). The full decision tree behind this classification is provided in Fig. S9, while the classification for each individual species is given in Table S16.

**Fig. 4:**
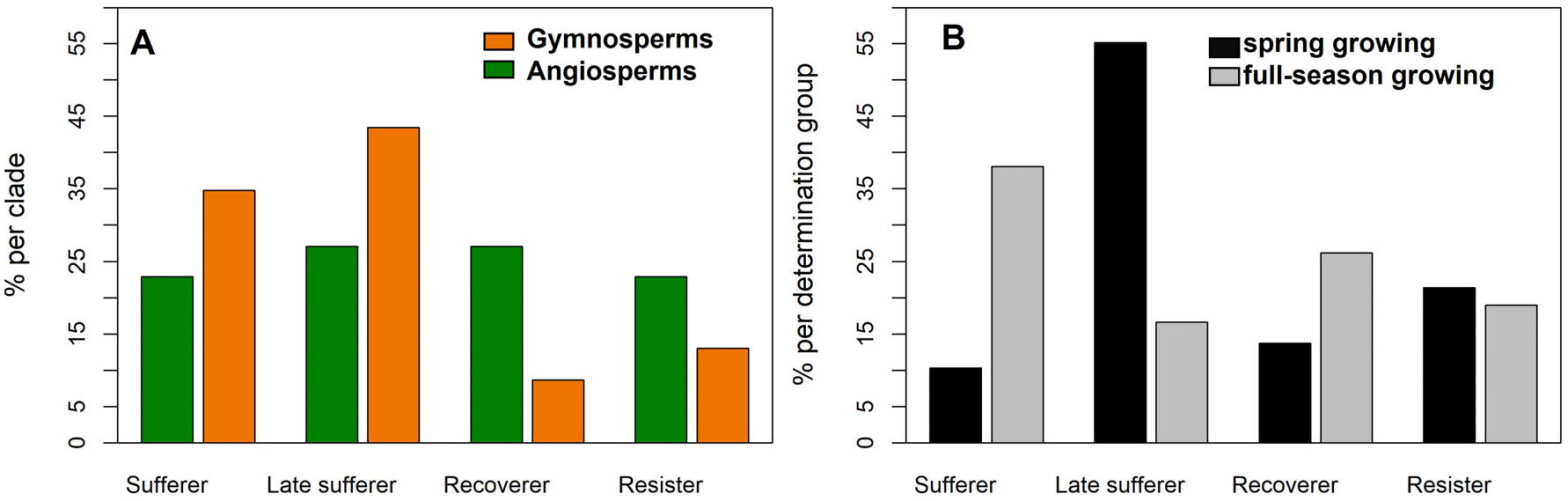
Distribution of (A) clades and (B) growth programmes into response types. (A) ‘Sufferer’, ‘Late sufferer’, ‘Recoverer’, and ‘Resister’ separated for clades of angiosperms and gymnosperms. Fisher's exact test showed significant differences between clades and response types (*p* = 0.149). (B) ‘Sufferer’, ‘Late sufferer’, ‘Recoverer’, and ‘Resister’ separated for groups of *spring growing* species and *full‐season growing* species. Fisher's exact test showed no significant differences between growth programmes and response types (*p* = 0.003).

We observed clear patterns in how these response types are distributed across the phylogenetic clades and that they are statistically independent of each other (Fig. 4A; *p* = 0.149). In the 23 gymnosperms, the growth patterns of ‘Sufferer’ (8 species) and ‘Late sufferer’ (10 species) were dominant, with only two ‘Recoverer’ and three ‘Resister’ species. Within the 48 angiosperms we found 11 ‘Sufferer’ and 13 ‘Late sufferer’, 13 ‘Recoverer’ and 11 ‘Resister’. As expected, for the growth programmes, the *spring growing* species were represented by the response type ‘Late sufferer’, while the *full‐season growing* species were predominantly ‘Sufferer’ and ‘Recoverer’, i.e. they responded earlier to the drought (Fig. 4B).

### Functional trait responses

**Fig. 5:**
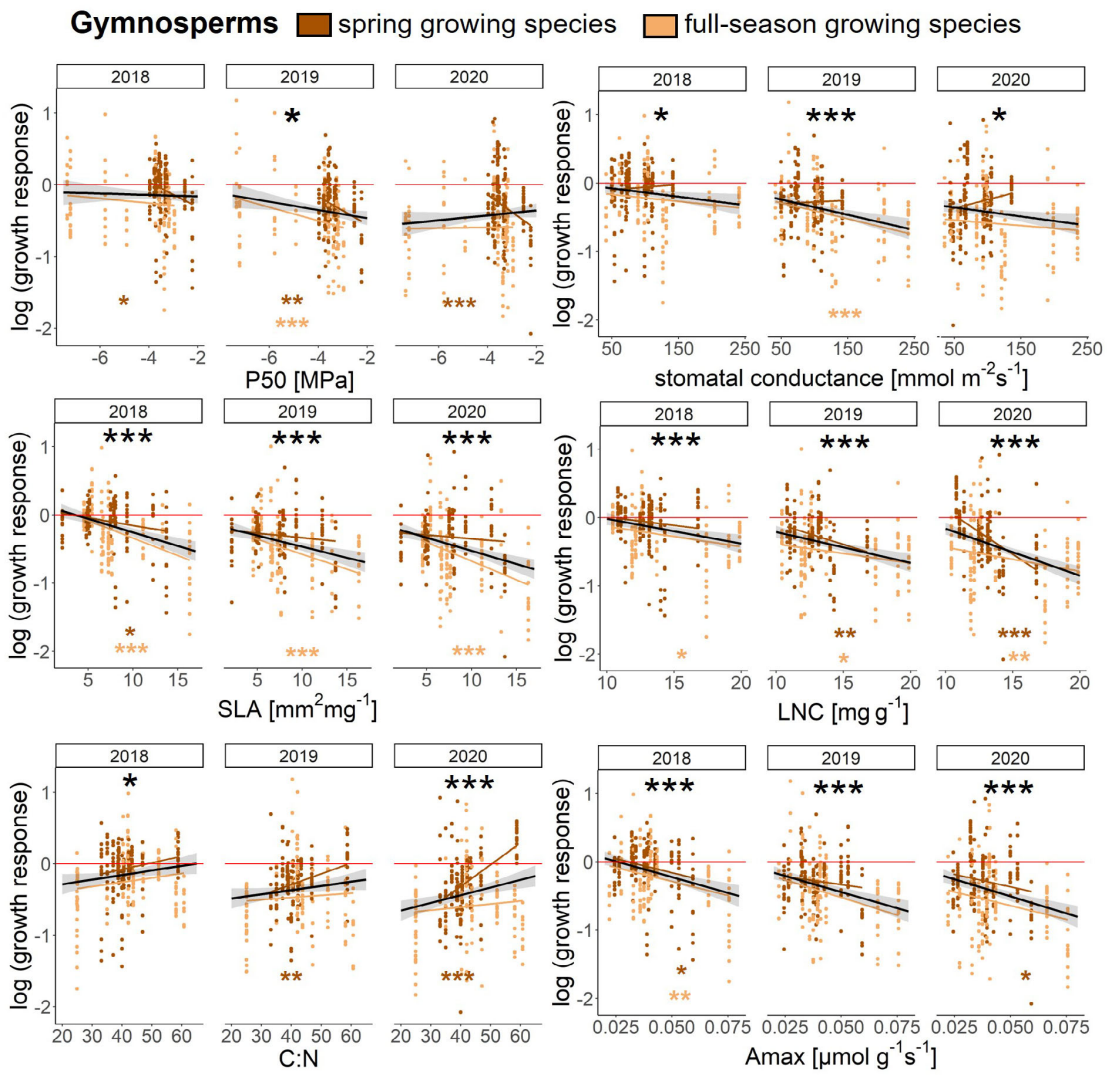
Trait‐driven responses in growth for gymnosperms during the 2018–2020 drought based on linear mixed‐effects model fits. Zero corresponds to comparable growth in drought and climatically normal years (mean of reference years 2016 and 2017), shown as a red zero‐line, while negative growth responses indicate growth reductions. Dark dots indicate *spring growing* species, while light dots indicate *full‐season growing* species. Black lines with 95% CI show overall trends; significant relationships indicated by black asterisks above linear mixed‐effect model fits (*p < 0.05, **p < 0.01, ***p < 0.001) within the year. Coloured asterisks below indicate significant relationships for each growth programme, respectively.

**Fig. 6:**
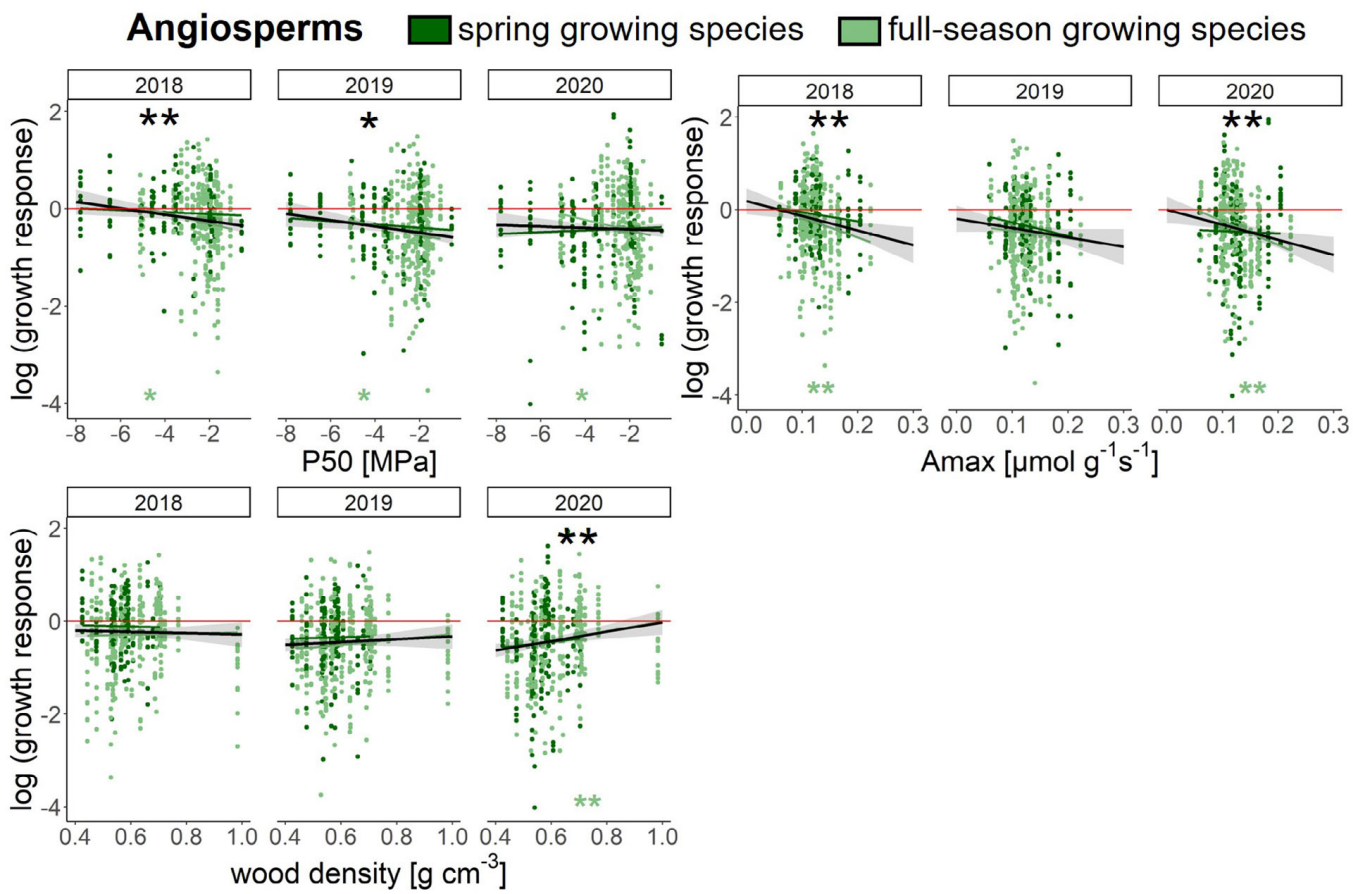
Trait‐driven responses in growth for angiosperms during the 2018–2020 drought based on linear mixed‐effects model fits. Zero corresponds to comparable growth in drought and climatically normal years (mean of reference years 2016 and 2017) shown as a red zero‐line, while negative growth responses indicate growth reductions. Dark dots indicate *spring growing* species, while light dots indicate *full‐season growing* species. Black lines with 95% CI show overall trends, and significant relationships are indicated by black asterisks above linear mixed‐effect model fits (*p < 0.05, **p < 0.01) within the year. Coloured asterisks below indicate significant relationships for each growth programme, respectively.

For the effects of the individual traits on growth responses to drought, we found significant evidence for relationships within both gymnosperms and angiosperms (Figs. 5, 6, S12; Tables S17, S18).

For gymnosperms, growth significantly decreased with increasing P50 (i.e. increased cavitation risk) in 2019 (Fig. 5; Tables S17 and S18), and also within the *full‐season growing* gymnosperms in 2019. *Spring growing* gymnosperms showed significantly decreasing growth with increasing P50 in all three years. With increasing stomatal conductance, growth of all gymnosperms decreased significantly in all three years. This pattern appeared to be driven by *full‐season growing* species (Fig. 5). Gymnosperm growth also decreased significantly with increasing SLA, LNC and A_max_, three functional traits associated with the LES, in all three years (Fig. 5; Table S17). Growth increased significantly with increasing C:N ratio in 2018 and 2020 across all species and for *spring growing* species in 2019 and 2020 (Fig. 5; Table S17). Growth responses were not significantly related to wood density across all gymnosperms (Fig. S12; Table S17). However, growth increased significantly with increasing wood density within the *spring growing* gymnosperms in all three years (Fig. S12; Table S17). These models explained between 17% and 32% of the variation in growth response, through their fixed effects (R^2^m) and 42%–50% through their fixed and random effects (R^2^c; Table S18).

For the angiosperms, growth decreased significantly with increasing P50 in 2018 and 2019 (Fig. 6; Table S17), also for *full‐season growing* but not for *spring growing* angiosperms in all three years. Stomatal conductance and SLA showed no significant relationships with growth responses for any angiosperms (Fig. S12). However, in 2020 *spring growing* and *full‐season growing* angiosperms showed opposite relationships for stomatal conductance and SLA, which most likely levelled each other out (Fig. S12, Table S17). Growth significantly decreased with increasing A_max_ for angiosperms and in *full‐season growing* angiosperms in 2018 and 2020, but not in *spring growing* angiosperms (Fig. 6). Growth increased significantly with increasing wood density in 2020 for all angiosperms and for *full‐season growing* angiosperms. These models explained 7%–10% of the variation in growth responses through their fixed effects (R^2^m) and 28%–30% through their fixed and random effects (R^2^c; Table S18).

### Trait spaces

**Fig. 7:**
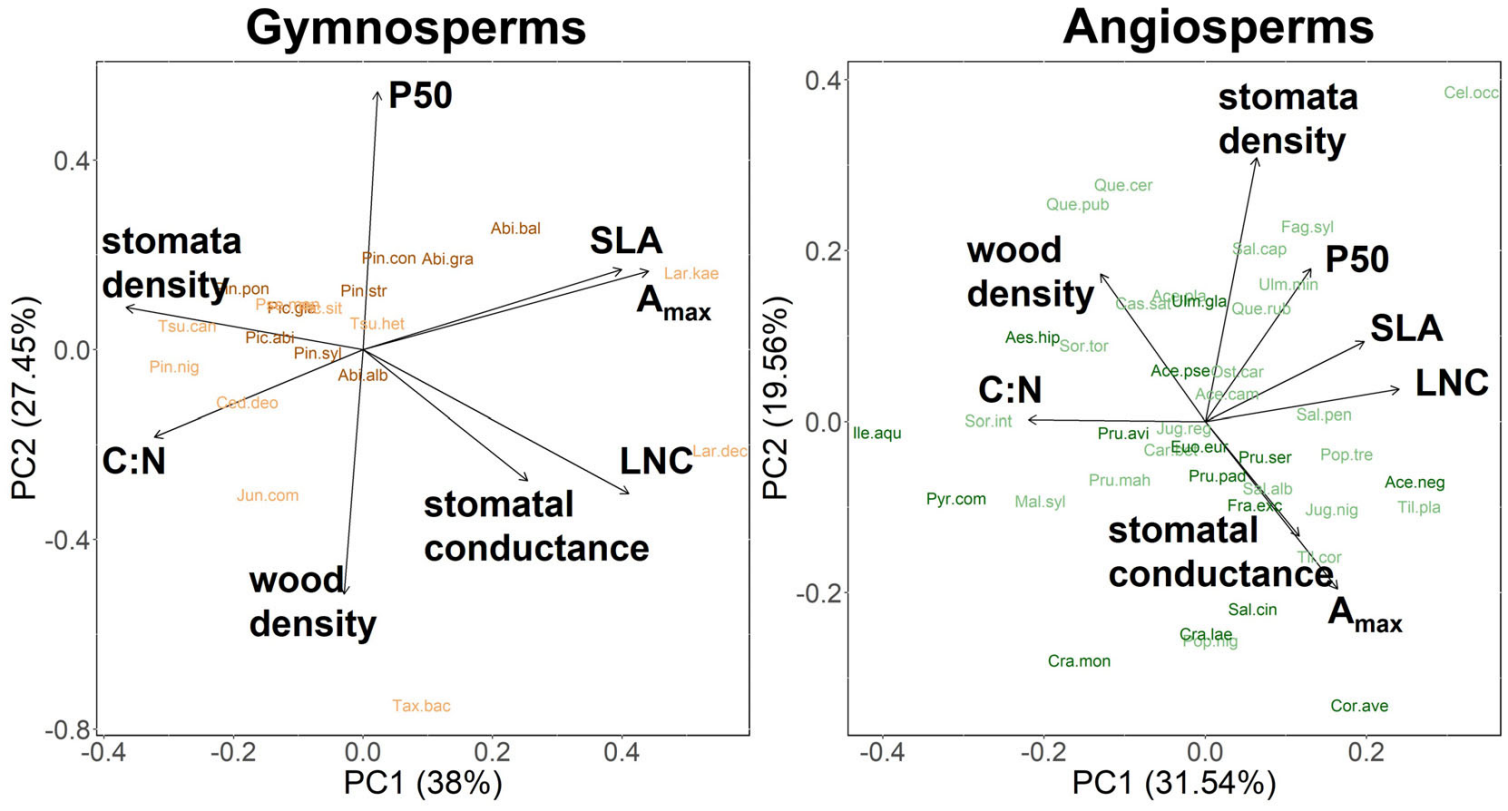
Principle components analyses (PCAs) of gymnosperms and angiosperms depicting the trait space of the continuous traits P50, stomatal density, stomatal conductance, SLA, LNC, C:N, A_max_, and wood density. Colours are related to the growth programme (Figs. 5 and 6).

Principal components analysis (PCA) of all species showed a clear separation between gymnosperms and angiosperms (Fig. S7). The main drivers were the traits: SLA, LNC, C:N and A_max_, which separated the two clades. Due to the strong separation in trait space between the clades, we ran the analyses separately for both clades (Table S19). For gymnosperms, we found that LES traits (SLA, LNC, C:N and A_max_) were mainly associated with the first PCA axis (PC1; 38% explained variation; Fig. 7), such that positive values represent an acquisitive trait expression (high SLA, A_max_ and LNC).

**Fig. 8:**
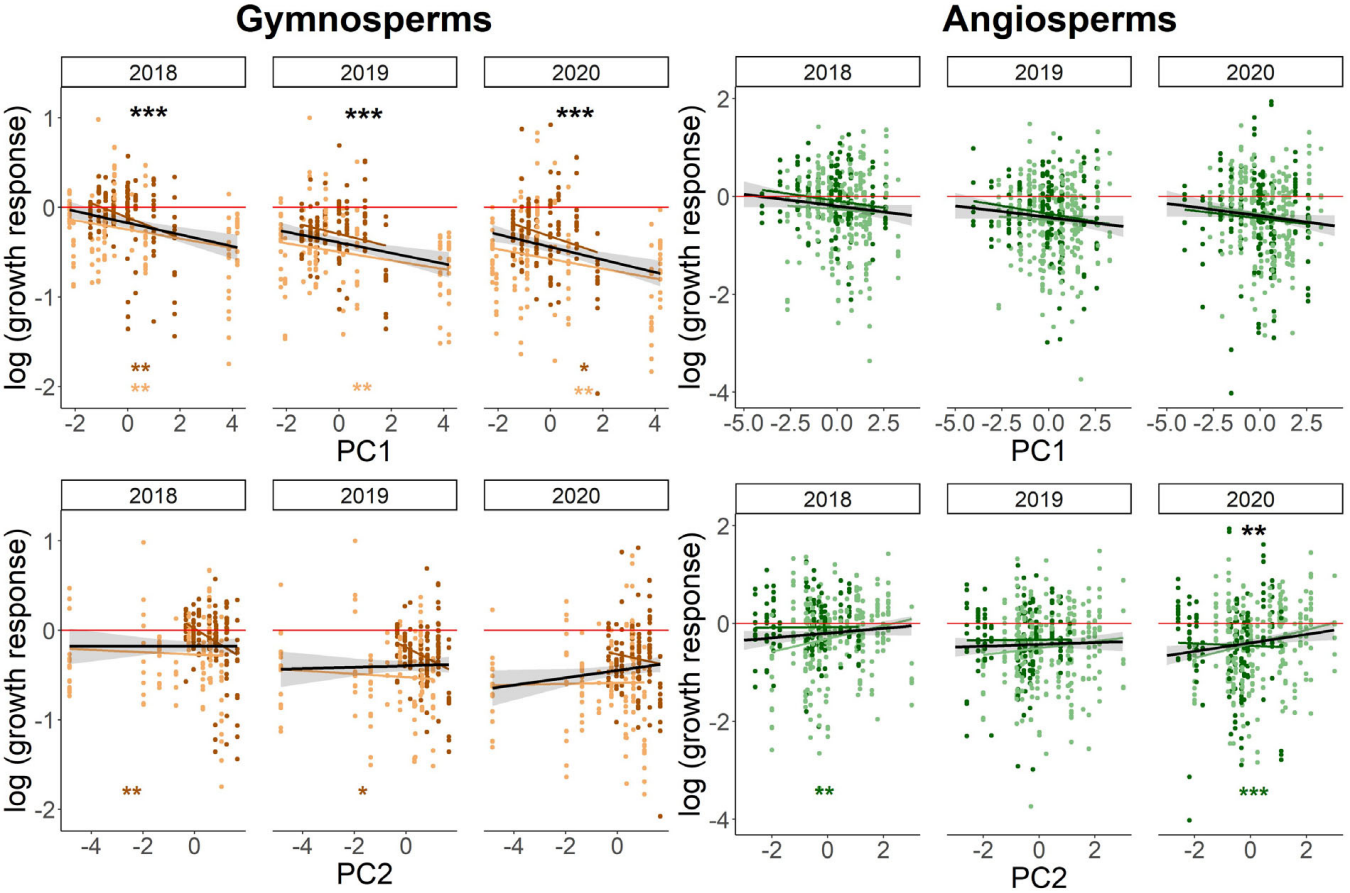
Principal components as predictors of tree growth response for gymnosperms and angiosperms. Shown are relationships between PC1 and PC2 and growth response of gymnosperm and angiosperm trees during the drought years 2018–2020 based on linear mixed‐effects model fits. Zero corresponds to comparable growth in drought and climatically normal years (mean of reference years 2016 and 2017) shown as a red zero line, while negative responses indicate growth reductions. Dark dots indicate *spring growing* species, while light dots indicate *full‐season growing* species. Black lines with 95% CI show overall trends, and significant relationships are indicated by black asterisks above linear mixed‐effect model fits (*p < 0.05, **p < 0.01, ***p < 0.001) within the year. Coloured asterisks below indicate significant relationships for each growth programme, respectively.

Using PC1 as a predictor for gymnosperm growth responses to drought, we found significantly decreasing growth with increasing PC1 values for all three drought years (R^2^m = 25%, R^2^c = 47%; Table S18), meaning that gymnosperms with acquisitive traits had lower growth during drought (Fig. 8, Table S17), consistent with the growth response to individual traits and also reflected in clear patterns for both growth programmes. For angiosperms, LES traits (SLA, LNC and C:N) were mainly associated with the first PCA axis (PC1, 31.54%; Fig. 7), but PC1 did not significantly affect growth responses of angiosperms (Fig. 8, Table S17).

For gymnosperms, the traits P50 and wood density formed a gradient in opposite directions, i.e. higher P50 was associated with lower wood density (PC2, 27.45%; Fig. 7). Growth decreased significantly with increasing PC2 values in 2018 and 2019, i.e. with decreasing cavitation resistance, but only for *spring growing* gymnosperms (Fig. 8). For the angiosperms, P50 and wood density both loaded on PC2 in a positive direction together with stomatal density. Growth increased with increasing PC2 values for all angiosperms in 2020 and, for *full‐season growing* angiosperms in 2018 and 2020. However, when PC4 was included, P50 and wood density clearly showed an opposite pattern (Fig. S8), similar to that found in responses of the individual traits (Fig. 6). For both clades, stomata density and stomatal conductance pointed in opposite directions, forming a gradient ranging from species with high stomatal density and low stomatal conductance to those with low stomatal density, but high conductance (PC1 in gymnosperms and PC2 in angiosperms; Fig. 7). Overall, we found no apparent pattern among the four response types within the clade trait spaces (Fig. S11). However, for angiosperms there were significant differences in growth programmes (MANOVA;*p* = 0.013).

## DISCUSSION

### Tree growth response to drought across species

During the three consecutive drought years 2018–2020, we observed significant tree growth reductions across 71 tree species growing at a single site under similar environmental conditions at the research arboretum ARBOfun. Overall, we confirmed our first hypothesis that the 2018–2020 drought caused growth reductions throughout the drought period, which were more pronounced in 2019 and 2020. During drought, trees lack water and face a trade‐off between carbon gain and water loss (Cowan & Farquhar 1977). Among other mechanisms, trees respond to this drought stress by reducing growth (McDowell *et al*. 2008; Adams *et al*. 2017; Schuldt *et al*. 2020; McDowell *et al*. 2022). In contrast to most previous studies, the use of shoot increment as a climate‐sensitive growth indicator allowed us to accurately measure tree growth at the organ level (as opposed to coarse inventories of height and diameter). Moreover, the trees at our study site are relatively young, and therefore the use of shoot increment was a suitable, non‐destructive alternative to taking tree cores for standard dendrochronological analyses (but see Liu *et al*. 2022).

Previous studies suggest that primary growth of leaf and shoot primordia and secondary growth of xylem are strongly correlated within trees (Cochard *et al*. 2005; Girard *et al*. 2012), and that species growth programmes should be taken into account in shoot increment analyses (Cochard *et al*. 2005; Savage & Chuine 2021). We accounted for growth programmes using weekly phenological observations at our study site.

We assume that most species we have classified as *spring growing* are species with preformed growth, although this has not been validated anatomically. In species with preformed growth, the number of phytomers (i.e. basic unit of a leaf, internodes and axillary bud) of shoots is already fixed in terminal buds formed in the previous year. In the growth year, the new shoot is completed through expansion growth within a short duration of about one to two month (Marks 1975; Brown & Sommer 1992). The influence of climate on growth is therefore limited by (i) the short duration of exposure to climate variation, and (ii) the climate which can influence size (albeit not number) of cells formed in a shoot. Although species with neoformed growth start with a set of preformed phytomers (Barthélémy & Caraglio 2007), meristematic activity responds to the current year's climate throughout the entire growing season by adding new phytomers. Indeed, the f*ull season growing* species tended to respond already in the first year of drought, 2018, and more strongly with growth responses across all years (Fig. 3). More than half of the *spring growing* species responded as ‘Late sufferer’ (Fig. 4B), and their growth was not significantly reduced in 2018 (Fig. 3). This suggests that they were able to complete their shoot growth in 2018 before the drought became more severe during the summer months. *Spring growing* species in both clades showed a larger reduction in growth from 2019 onwards, most likely due to the extreme climate in 2018, which resulted in shorter preformed shoots. Cochard *et al*. (2005), studying beech trees, found indications that reduced secondary growth reduces xylem water transport and thus bud growth for the next season. Legacy effects, such as damage to the tree's water transport system, are affect trees and forests up to 5 years after the drought (Girard *et al*. 2012; Anderegg *et al*. 2015; Kannenberg *et al*. 2018; Zweifel & Sterck 2018; Schnabel *et al*. 2022). It is possible that preformed growth, that is, execution of a predefined growth programme, limits the tree's ability to adaptively downregulate shoot growth and shift allocation to belowground organs under adverse climate conditions. This, in turn, could lead to increased stress during the growth year and, consequently, increased expression of a legacy effect in subsequent years. However, we observed weaker legacy effects in *spring growing* species (Fig. 3). More immediate responses could also help *full‐season growing* species to recover earlier. In angiosperms, this could explain the moderate recovery of growth in 2020—a year with slightly better growing conditions (Fig. 3E).

Growth of gymnosperms was more strongly reduced during drought than that of angiosperms (Fig. 3). This is supported by the fact that >75% of the gymnosperm species suffered during the consecutive drought years and were therefore classified as either ‘Sufferer’ or ‘Late sufferer’. In contrast, only 50% of the angiosperms were classified in these response types (Fig. 4A, Table S16). Other studies observed a similar pattern, i.e. that gymnosperms suffer more strongly during drought, as reinvestment in damaged leaves is costly for species with a longer leaf lifespan, such as gymnosperms (Anderegg *et al*. 2020; Song *et al*. 2022; Sangüesa‐Barreda *et al*. 2023). However, equally high mortality risks during drought were found for angiosperms and gymnosperms worldwide (Anderegg *et al*. 2016), but growth responses prior to mortality were not included in this study. We observed strong differences in explained variation by fixed and random effects between species, which may be related to differences in climate sensitivity and intraspecific variability.

### Drought‐tolerance traits can explain growth response

For the drought‐tolerance trait P50, there were significant growth reductions with increasing P50 (i.e. increasing cavitation sensitivity) in gymnosperms and angiosperms (Figs. 5, 6). Interestingly, the signal was consistent across all three drought years for *spring growing* gymnosperms and *full‐season growing* angiosperms. Stronger signals in *full‐season growing* angiosperms are generally expected due to their greater capacity to respond to climate variability, while the signal in *spring growing* gymnosperms requires further investigation. Overall, we were able to confirm our third hypothesis, as species with functional traits expressions indicative of high drought tolerance, here represented by lower (more negative) P50 values, showed less growth reduction (Petruzzellis *et al*. 2022).

For *full‐season growing* angiosperms, increasing wood density was related to increased growth in 2020, as expected. For all gymnosperms there were no overall relationships, but for only *spring growing* gymnosperms wood density, which in tracheid‐composed wood is negatively related to conduit size (Rathgeber *et al*. 2006) and thus cavitation risk, was positively related to growth in all three years. This is indirectly supported by the fact that in gymnosperms, wood density was negatively related to P50 (PC2; Fig. 7). Similarly, wood density and P50 were negatively related in angiosperms (albeit on PC4; Fig. S8). Thus, a low P50, that is, high resistance to cavitation, coincides with high wood density, which should bolster growth response. This supports previous evidence that wood density is associated with other drought‐tolerance traits, such as hydraulic safety margin and P50 (Rosner 2017; Oliveira *et al*. 2021). Although the overall effect of wood density on growth response to drought is still debated (Poorter 2008; Chave *et al*. 2009), our study provides evidence that wood density is associated with increased growth resistance.

For gymnosperms and angiosperms, stomatal density and stomatal conductance loaded in opposite directions in the PCAs (Fig. 7), suggesting that species with low stomatal density have high stomatal conductance, most likely related to few but large stomata. As hypothesized (Hypothesis 3), gymnosperms with lower stomatal conductance had a higher growth. Furthermore, for gymnosperms, stomatal conductance loaded in a similar direction as LNC, SLA and A_max_ (PC1, Fig. 7), an association previously reported, albeit for angiosperms in the subtropics (Kröber *et al*. 2014). Thus, we expect a high stomatal conductance to be associated with acquisitive resource use. However, for angiosperms, we did not find such an association between stomatal traits and LES traits, nor with the hydraulic trait P50, which could be explained by the significant interaction between growth programmes and stomatal conductance, and the contradictory trends between *spring growing* angiosperms and *full‐season growing* angiosperms (Fig. S12; Table S17).

### Leaf Economics Spectrum traits can explain growth response

In gymnosperms, LES trait expressions, associated with conservative resource use, were associated with stronger growth reductions during drought (Fig. 5; Table S17). In addition, these LES traits formed an important axis of functional variation on the first PCA axis (i.e. SLA, LNC, C:N and A_max_), ranging from slow to fast strategies. As expected, PC1 was negatively related to the growth response in all three drought years (Fig. 8; Table S17). These trait–growth relationships were consistent across the two growth programmes. For angiosperms, there were significantly negative growth responses to A_max_ in 2018 and 2020, which were predominately driven by *full‐season growing* angiosperms (Fig. 6; Table S17), showing that species with high light‐saturated maximum photosynthesis rates—usually associated with rapid growth—have low growth under drought. Furthermore, for angiosperms, most of the LES traits loaded on the first PCA axis (i.e. SLA, LNC and C:N; Fig. 7). Thus, we confirmed hypothesis 4 for gymnosperms and angiosperms (although weaker associations of LES traits were observed in angiosperms). Consequently, species with resource acquisition traits favouring rapid growth are more susceptible to drought and thus show a larger reduction in growth during drought. Previous studies have suggested that traits related to conservative resource use and slow growth strategies are associated with: (1) a lower drought‐related tree mortality across biomes (Greenwood *et al*. 2017), and (2) a higher drought tolerance (Guillemot *et al*. 2022, although based on tropical tree species). Similar to our findings, a recent study in subtropical experimental tree communities reported that acquisitive species had lower growth under drought conditions than cavitation‐resistant species (Schnabel *et al*. 2024), albeit based on fewer tree species. The weaker trend for the angiosperms in our study may be because 50% of the angiosperms did not suffer substantially during the entire drought period (Fig. 4A; Table S16). Furthermore, our study shows that although there was a legacy effect in the growth response in 2019 and 2020, control through the LES traits was directly present from the first drought year onwards. For the first time, we report clear evidence that LES traits control tree growth responses to drought across a wide set of species under nearly identical growing conditions and successive hotter drought conditions.

### Climate‐smart forest management

Trees in the ARBOfun arboretum were planted at a wide spacing, which prevented tree–tree interactions. Thus, our results can be interpreted as the intrinsic, trait‐driven responses of the species to climate conditions without the effect of competition or facilitation (Forrester & Pretzsch 2015), which are otherwise present in forests and shape the effects of functional traits on ecosystem functioning (Trogisch *et al*. 2021). Our study thus captures ‘pure’ trait‐driven responses of a broad range of Central European tree species to consecutive drought years, species that dominate forests today, but importantly, also those that may dominate under future climate regimes. The traits and trait syndromes (such as P50 and the LES) observed to influence growth response to drought can be used to inform management decisions on tree species selection and to improve the predictive capacity of forest models. Nevertheless, it is important to note that we studied young trees and that growth responses to drought may differ in older trees growing in mature forests. The identification of the four response types helped to account for varying growth response patterns across species, but also provided important insights for individual key species. For example, the two currently most economically important tree species in Central European managed forests, *P. abies* and *P. sylvestris*, which together account for 47.7% of Germany's managed forests (BWI, 2012), suffered strongly in recent years (Senf *et al*. 2020). They showed a drought response of ‘Late sufferer’, indicating that they are likely to suffer strongly from more frequent and intense drought in the coming century (IPCC 2014). In contrast, the angiosperms *F. sylvatica* and *Q. robur*, which currently comprise 25.8% of Germany's managed forests (BWI, 2012), showed response types of ‘Recoverer’ and ‘Resister’, respectively. Buras & Menzel (2019) and Kölling & Mette (2022) also classified these two species as more resistant to climate change. While the drought resistance of *F. sylvatica* is controversial (Kunz *et al*. 2018), we identified this species as ‘Recoverer’. Two species with low commercial value at present, but with the potential to gain economic importance for Central European forests in the future are *S. torminalis* and *Q. pubescens* (Kunz *et al*. 2018; Buras & Menzel 2019). We also classified these two species as the response types ‘Recoverer’ and ‘Resister’, respectively. Thus, our response type classification approach can help to understand responses of individual species to drought. That we did not find clear patterns of the response types within the trait spaces (Fig. S11), suggests that similar responses can be achieved by different but equivalent trait configurations, which warrants further investigation.

### Reflection

We deliberately did not correct for phylogeny because the separation of clades (angiosperms and gymnosperms) already captured much of the phylogenetic signal (see Fig. S7). As we examined even‐aged trees, we did not control for tree size. However, drought resistance was found to decrease with tree height (McGregor *et al*. 2021). Moreover, growth programmes may change with age for individual species, as some species may evolve from species with neoformed buds to species with preformed buds as they mature (Gordon *et al*. 2006). The gap‐filling of the trait data from the TRY database is a helpful and indispensable tool for investigating many traits for a wide set of species. It has been found that global trait data, such as TRY data, are valid for explaining local patterns, as inter‐specific variation is typically greater than intra‐specific variation (Kazakou *et al*. 2014). Furthermore, P50 in particular, which is an important drought‐tolerance trait (Choat *et al*. 2012), is difficult to measure, especially in ring‐porous species with very long vessels. Therefore, we excluded P50 values >−0.5, as suggested by Sergent *et al*. (2020), due to unrealistically high values. Overall, our trait‐based models explained only moderate variation in growth responses, with a higher predictive capacity for gymnosperms compared to angiosperms (Table S18). We expect that with more and especially in‐situ measured traits, such models will likely increase in their predictive capacity. Similarly, with such improved trait coverage, we may eventually be able to derive trait‐based predictions for assigning species to the observed response types.

## OUTLOOK AND CONCLUSION

For future studies, we plan to measure functional traits in‐situ, which should potentially improve growth predictions under consecutive hotter droughts. In addition to the drought‐tolerance and LES traits studied here, other hydraulic traits, such as turgor loss point or hydraulic safety margin, and belowground traits may be important predictors of growth response to drought. Particularly belowground traits, such as specific root length, root tissue density or root C:N, which capture a conservation, collaboration and plant size gradient (Comas *et al*. 2013; Bergmann *et al*. 2020; Weigelt *et al*. 2021), have already been found to influence above‐ and belowground plant productivity under drought (Comas *et al*. 2013; Brunner *et al*. 2015). The importance of drought‐tolerance and LES traits for tree growth responses to drought, and the recovery of some species under consecutive droughts, suggests that functional traits could also explain growth resilience. Some species, such as *F. sylvatica*, *Q. rubra* or *S. torminalis*, already recovered during the drought, although an overarching legacy effect was evident. However, we do not know if or when the species of ‘Sufferer’, such as *Larix decidua* or *Ulmus laevis*, and ‘Late sufferer’, such as *Acer campestre*, *P. abies* or *P. sylvestris*, will recover over time. Therefore, investigating growth resilience and recovery from recurrent and prolonged drought is of great interest at our study site. In future, we plan to examine tree growth expression during pre‐drought, drought and post‐drought years, to further explore the underlying, trait‐based mechanisms by incorporating in‐situ measured trait data that capture both above‐ and belowground trait gradients. Our work has shown that the internal growth programme influences the temporal patterns of drought responses and trait–growth relationships. We present the first study to systematically investigate this boundary condition. In future, it will be important to consider this effect, but also the biological consequences of these fundamentally different growth programmes for climate sensitivity and even drought‐related mortality.

To conclude, we observed significantly reduced growth in 71 tree species during the consecutive hotter drought years 2018–2020, with legacy effects further reducing growth in 2019 and 2020. Drought‐tolerance and LES traits were important predictors of growth responses, with stronger growth reductions observed in species with trait expressions indicative of low drought tolerance (high P50), rapid growth and acquisitive resource use (high SLA, LNC, and A_max_). Trait–growth relationships were clearer in gymnosperms than in angiosperms, and they differed predictably between growth programmes (*spring growing* species vs *full‐season growing* species). We expect that these findings will facilitate the development of forest management strategies under a future climate regime, characterised by more frequent, severe and prolonged droughts, through supporting tree species selection and improving forest models.

## Author Contributions

LK: Formal analysis, visualization, writing – original draft preparation; FS: Conceptualization, formal analysis, methodology, supervision, writing – review and editing; RR: Conceptualization, formal analysis, writing – review and editing; AR: Investigation, writing –review and editing; JK: methodology, formal analysis, writing – review and editing; KA: Formal analysis, writing – review and editing; AK: Investigation, writing – review and editing; TK: Investigation, writing – review and editing; CW: Conceptualization, supervision, funding acquisition, project administration, writing – review and editing.

## TRY data citation

Adler *et al*. 2014; Adriaenssens 2012; Anderson *et al*. 2020; Atkin *et al*. 2015; Aubin *et al*. 2012; Benomar *et al*. 2018; Bjorkman *et al*. 2018; Campetella *et al*. 2011; Castro‐Díez *et al*. 1998; Chave *et al*. 2009; Chen *et al*. 2014; Chen *et al*. 2013; Choat *et al*. 2012; Coomes *et al*. 2008; Cornelissen 1996; Cornelissen *et al*. 2003, 2004; Cornwell *et al*. 2008; Craine *et al*. 2009; Dechant *et al*. 2017; Diaz *et al*. 2004; Dong *et al*. 2017, 2020; Douma *et al*. 2012; E‐Vojtkó *et al*. 2020; Falster *et al*. 2016; Feng & van Kleunen 2014; Findurová 2018; Fitter & Peat 1994; Freschet *et al*. 2010; Garnier *et al*. 2007; Han *et al*. 2005, 2012; Heberling & Mason 2018; Helsen *et al*. 2020; Kattge *et al*. 2009; Kerkhoff *et al*. 2006; Kleyer *et al*. 2008; Knauer *et al*. 2018; Laughlin *et al*. 2010, 2011; Liebergesell *et al*. 2016; Lin *et al*. 2015; Lukeš *et al*. 2013; Maire *et al*. 2015; Manzoni *et al*. 2013; Medlyn *et al*. 1999; Meir *et al*. 2002; Michaletz & Johnson 2006; Milla & Reich 2011; Mori *et al*. 2015; Niinemets 2001; Nolan *et al*. 2018; Núñez‐Florez *et al*. 2019; Ogaya & Peñuelas 2003; Olson *et al*. 2020; Olson *et al*. 2018; Onoda *et al*. 2017; Ordoñez *et al*. 2010; Paine *et al*. 2015; Pan *et al*. 2019; Pausas *et al*. 2016; Perea *et al*. 2021; Poorter *et al*. 2009; Prentice *et al*. 2011; Preston *et al*. 2006; Price & Enquist 2007; Quested *et al*. 2003; Raevel *et al*. 2012; Reich *et al*. 2008, 2009; Royer‐Tardif *et al*. 2018; Sack *et al*. 2003, 2006; Sancho‐Knapik *et al*. 2021; Scherer‐Lorenzen *et al*. 2007; Smith *et al*. 2016; Smith & Dukes 2017; Soudzilovskaia *et al*. 2013; Tang *et al*. 2013; Tavşanoğlu & Pausas 2018; Torres‐Ruiz *et al*. 2017; Vergutz *et al*. 2012; Wagenführ 2007; Walker 2014; Wang *et al*. 2018; White *et al*. 2000; Willis *et al*. 2010; Wilson *et al*. 2000; Wirth & Lichstein 2009; Wright *et al*. 2004; Yguel *et al*. 2011; Zanne 2009.

## Supporting information


**Data S1.** Supporting Information.
