# Supplementary material for: Functional traits explain growth response to successive hotter droughts across a wide set of common and future tree species in Europe
Source: Plant Biol (Stuttg). 2025 May 7;28(3):827–45. doi: 10.1111/plb.70024 (PMC13089614; doi:10.1111/plb.70024)
Supplement: Supplementary file 1 — Data S1. Supporting Information. [file PLB-28-827-s001.docx]

**Supporting Information**

Equation S1

1. Model_growth_response <- lme(log(growth_response) ~ as.factor(year), random= ~ 1 | tree_ID/branch_ID, correlation = corAR1(form = ~ as.numeric(year)), data = data, na.action= na.exclude)
2. model_growth-program<-lme(log(growth_response) ~ growth-program * as.factor(year), random= ~ 1 | tree/branch, correlation = corAR1(form = ~ as.numeric(year)), data = data, na.action= na.exclude)
3. model_SLA_growth-program<-lme(log(growth_response) ~ SLA * as.factor(growth-program) *as.factor(year), random= ~ 1 | tree/branch, correlation = corAR1(form = ~ as.numeric(year)), data = data, na.action= na.exclude)

**Figure S2**

**
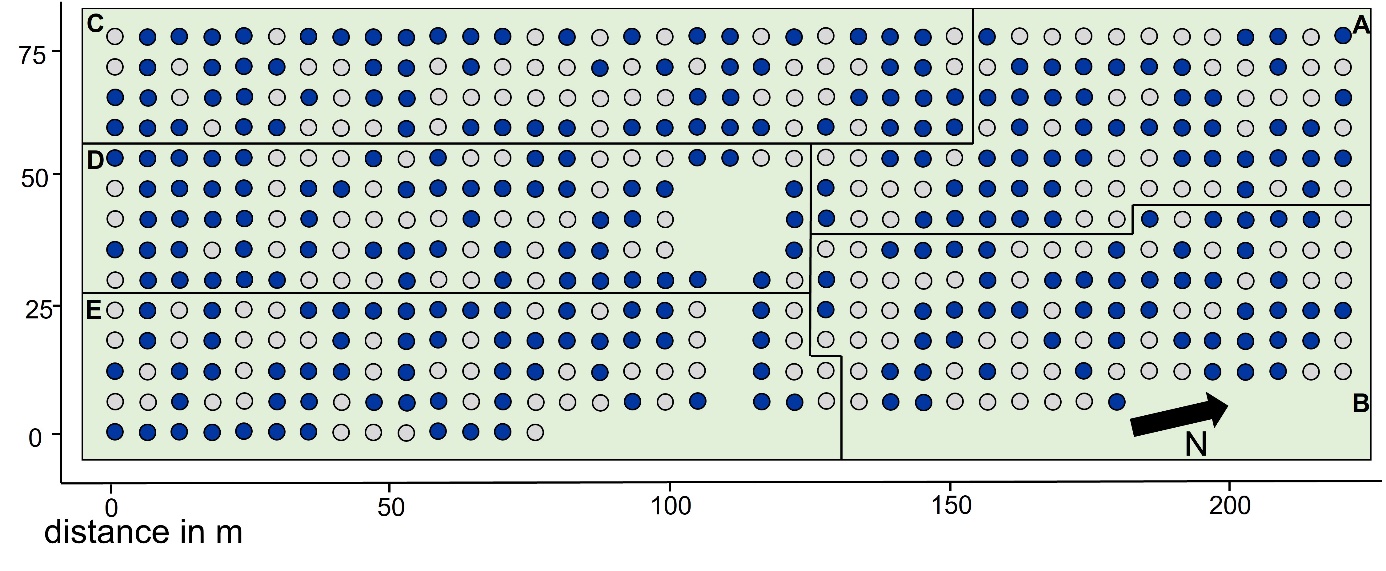
**

**Figure S2**: Study site. Top view on the ARBOfun research arboretum. The points represent the 100 species, each randomized within 5 blocks (A-E). The dark blue colour marks the trees used for this study. The planting distance between trees is 5.8 m.

Figure S3

**
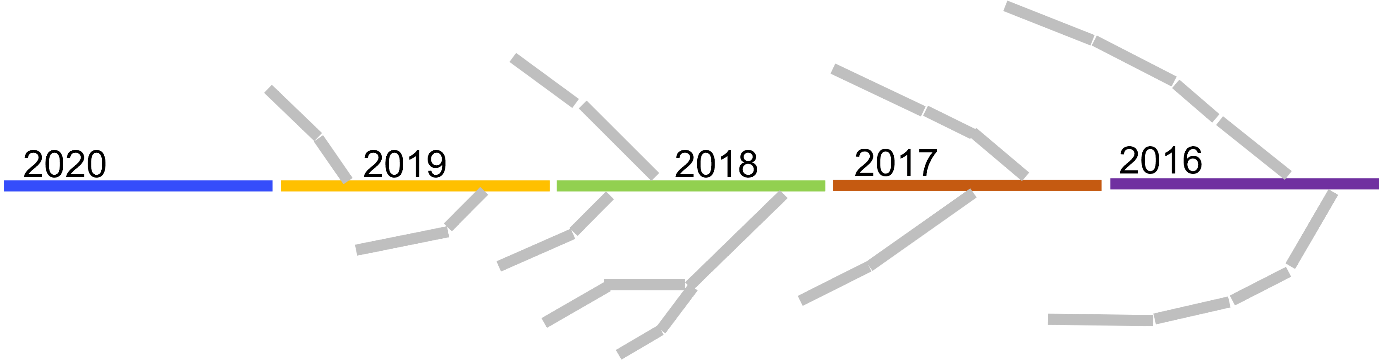
**

**Figure S3:** Sketch of a branch showing the shoot increment per year back to 2016. Shoot increment was always measured at the longest lateral branch.

Figure S4

**
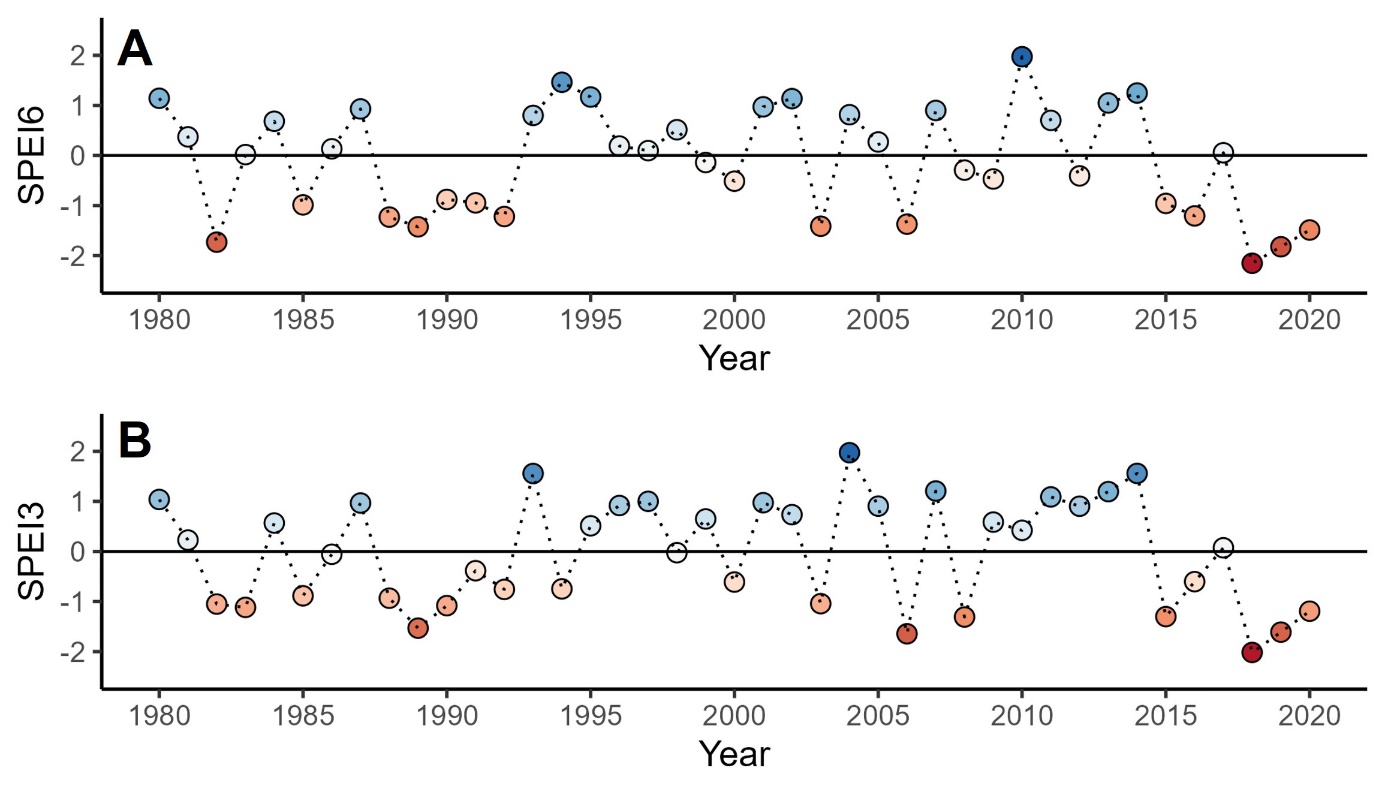
**

**Figure S4**: Standardised Precipitation-Evapotranspiration Index (SPEI) calculated for six (SPEI6) and three month (SPEI3). The SPEI6 was calculated for the six months of the vegetation period (April to September) for each year. The SPEI3 was calculated for the three months of the peak vegetation period (May to July) for each year. The zero line is the reference period 1981–2010. Blue-coloured dots indicate positive SPEI values, while red-coloured dots show negative SPEI values. SPEI values below -1 indicate exceptionally dry conditions, while values above 1 suggest exceptionally wet conditions (McKee et al. 1993).

Figure S5


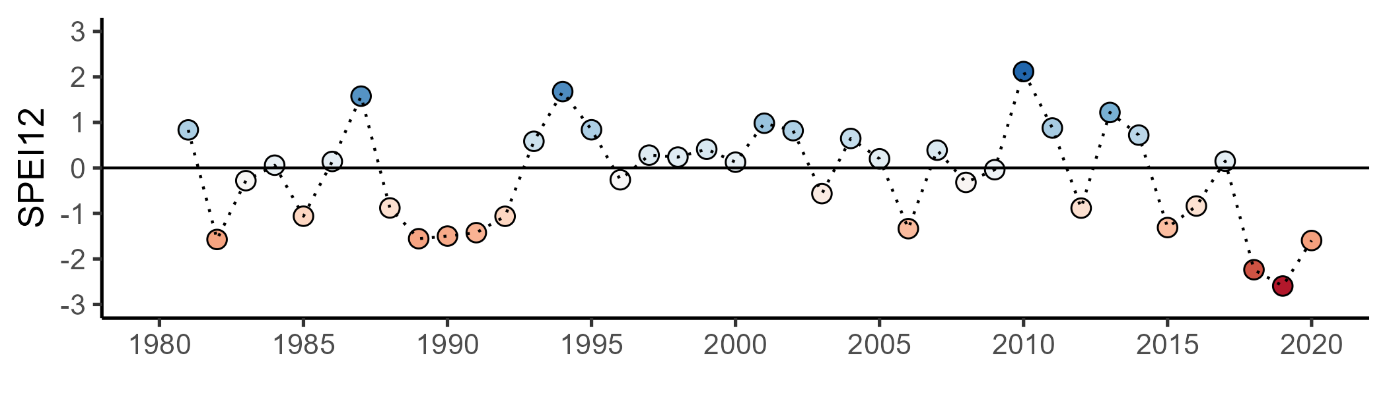
 **Figure S5**: Standardised Precipitation-Evapotranspiration Index (SPEI) calculated for 12 month period from September to August. The zero line is the reference period 1981–2010. Blue-coloured dots indicate positive SPEI values, while red-coloured dots show negative SPEI values.

Figure S6


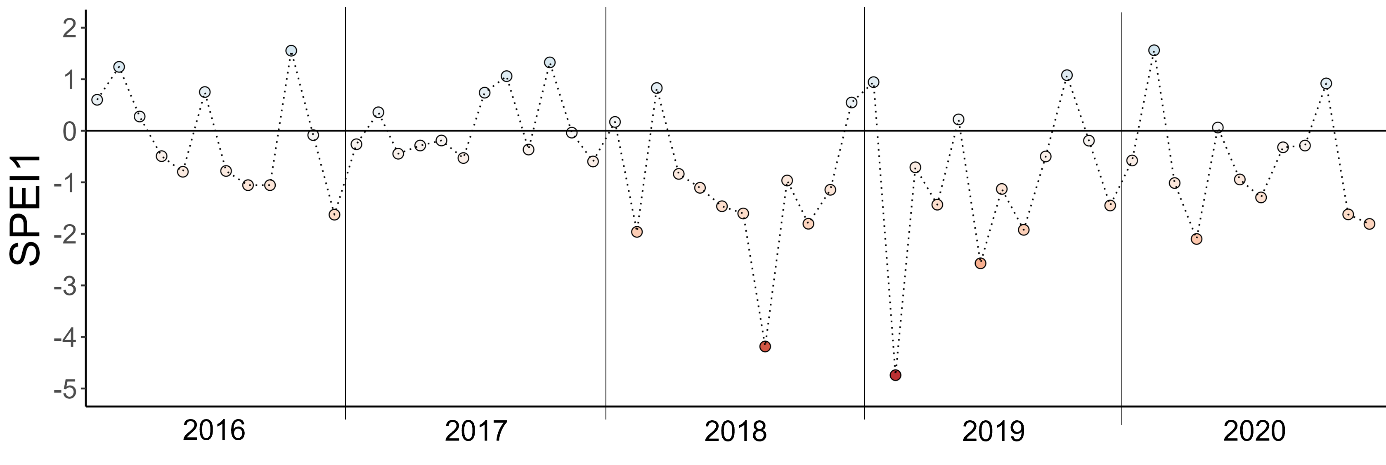


**Figure S6**: Standardised Precipitation-Evapotranspiration Index (SPEI) calculated per month (SPEI1) for the years 2016–2020. The zero line is the reference period 1981–2010. Blue-coloured dots indicate positive SPEI values, while red-coloured dots show negative SPEI values. SPEI values below -1 indicate exceptionally dry conditions, while values above 1 suggest exceptionally wet conditions (McKee et al. 1993), that is monthly SPEI values can single the start of a drought.

Figure S7


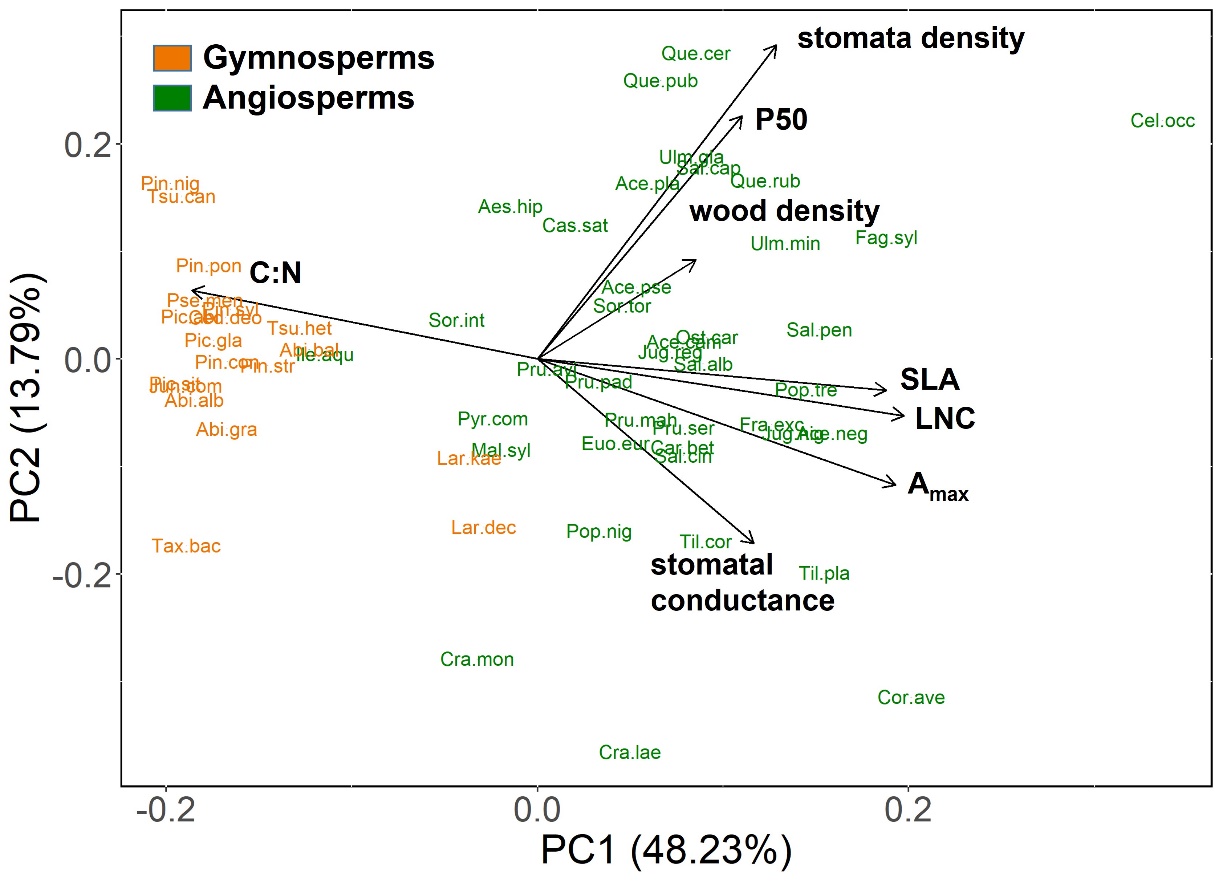


**Figure S7**: PCA of all species depicting the trait space of the continuous variables P50, stomatal density, stomatal conductance, SLA, LNC, C:N, A_max_, and wood density.

Figure S8


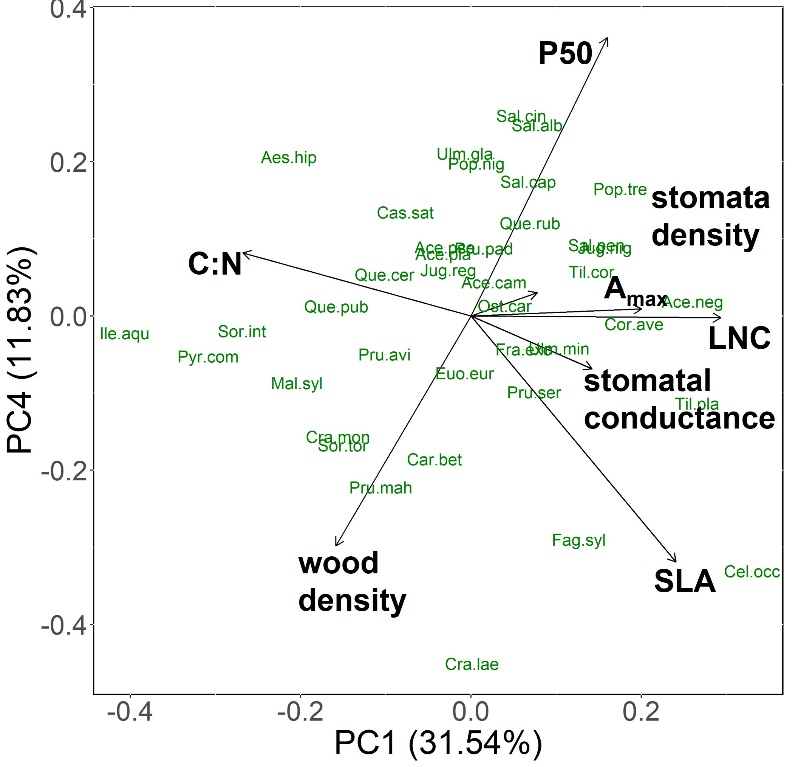


**Figure S8**: PCA of angiosperms (PC1 and PC4) depicting the trait space of the continuous traits P50, stomatal density, stomatal conductance, SLA, LNC, C:N, A_max_, and wood density.

Figure S9


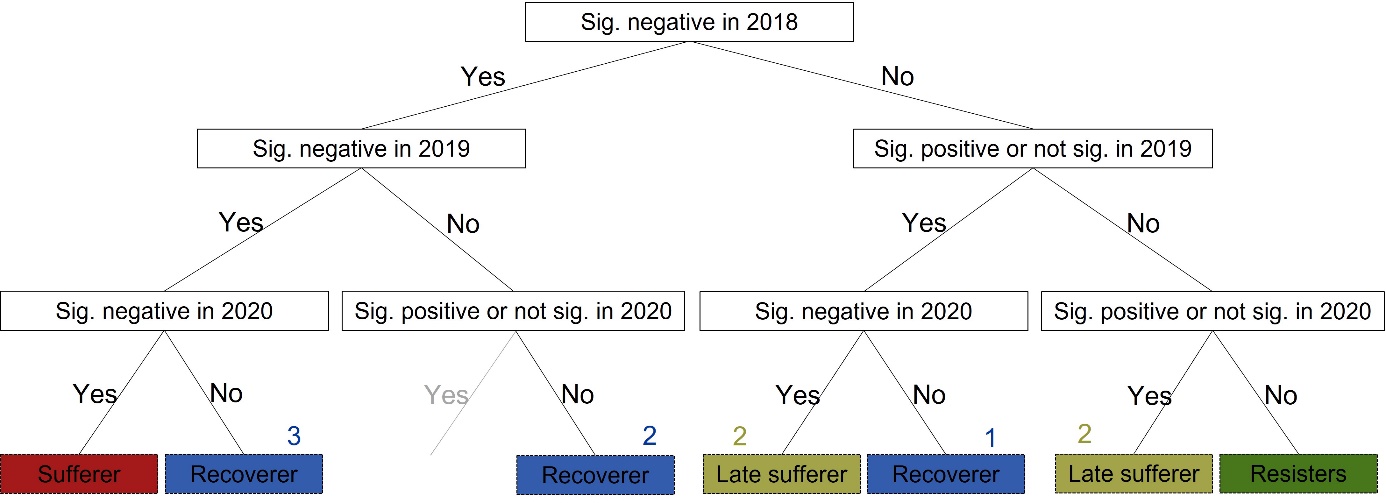


**Figure S9**: Decision tree for the classification of the response types, based on the linear mixed-effect models of the single species. The final classification due to the four response types ‘Sufferer’, ‘Late sufferer’, ‘Recoverer’ and ‘Resisters’ shows the reaction patterns of growth responses during the three drought years. The numbers next to the yellow (Late sufferer) and blue boxes (Recoverer) link the paths to the growth response pattern in Figure S10. The grey line did not occur for any of the investigated species.

Figure S10


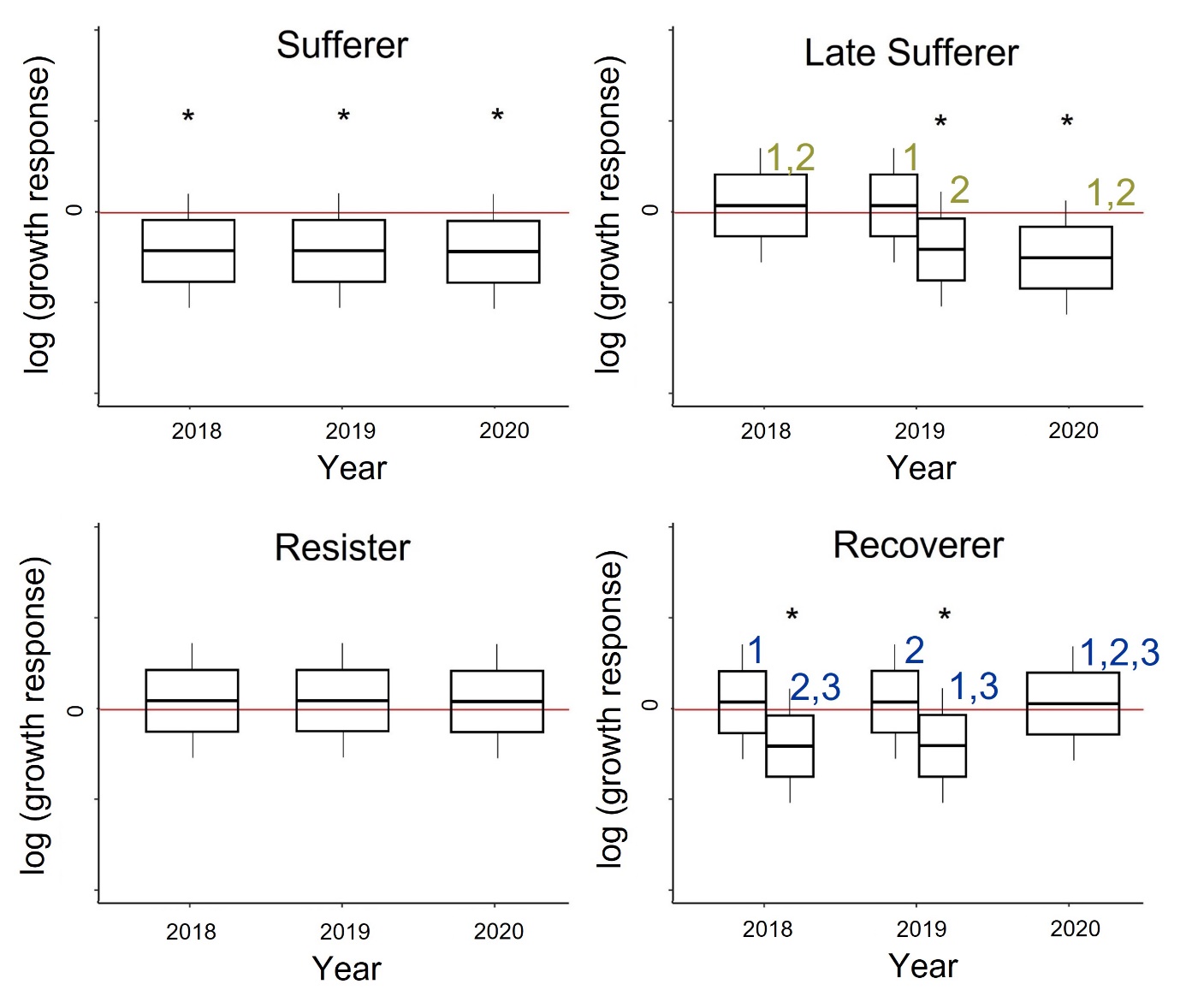


**Figure S10**: Response type classification. Growth response patterns over the three drought years 2018–2020 for the four response types classified due to the decision tree in Figure S9. The boxplots above the red reference zero line represent positive or not significant response and the ones below the red zero line negative response values with asterisks indicating a significant growth reduction. The divided boxplot for the Late sufferer in 2019 and for the Recoverer in 2018 and 2019 show positive and not significant (1^st^ boxplot) or significant negative (2^nd^ boxplot) effects, since they represent two divergent paths of the decision tree (Figure S9). For the Recoverer, either 2018 or 2019 or both years needed to be significantly negative as shown in the decision tree (Figure S9). The coloured numbers for Late sufferer and Recoverer link to the specific decision paths in the decision tree (Figure S9).

Figure S11

**
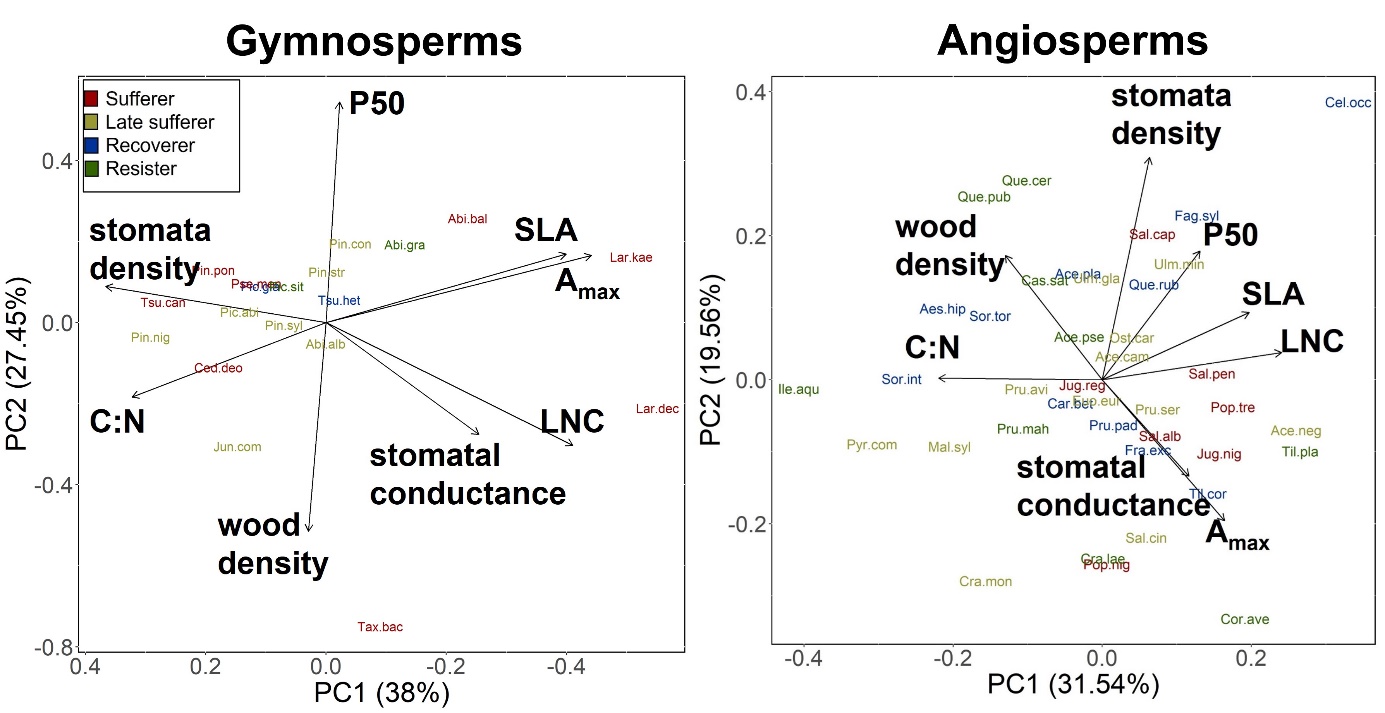
**

**Figure S11**: PCAs of gymnosperms and angiosperms depicting the trait space of the continuous variables P50, stomatal density, stomatal conductance, SLA, LNC, C:N, A_max_, and wood density. Colours are due to the response types.

Figure S12


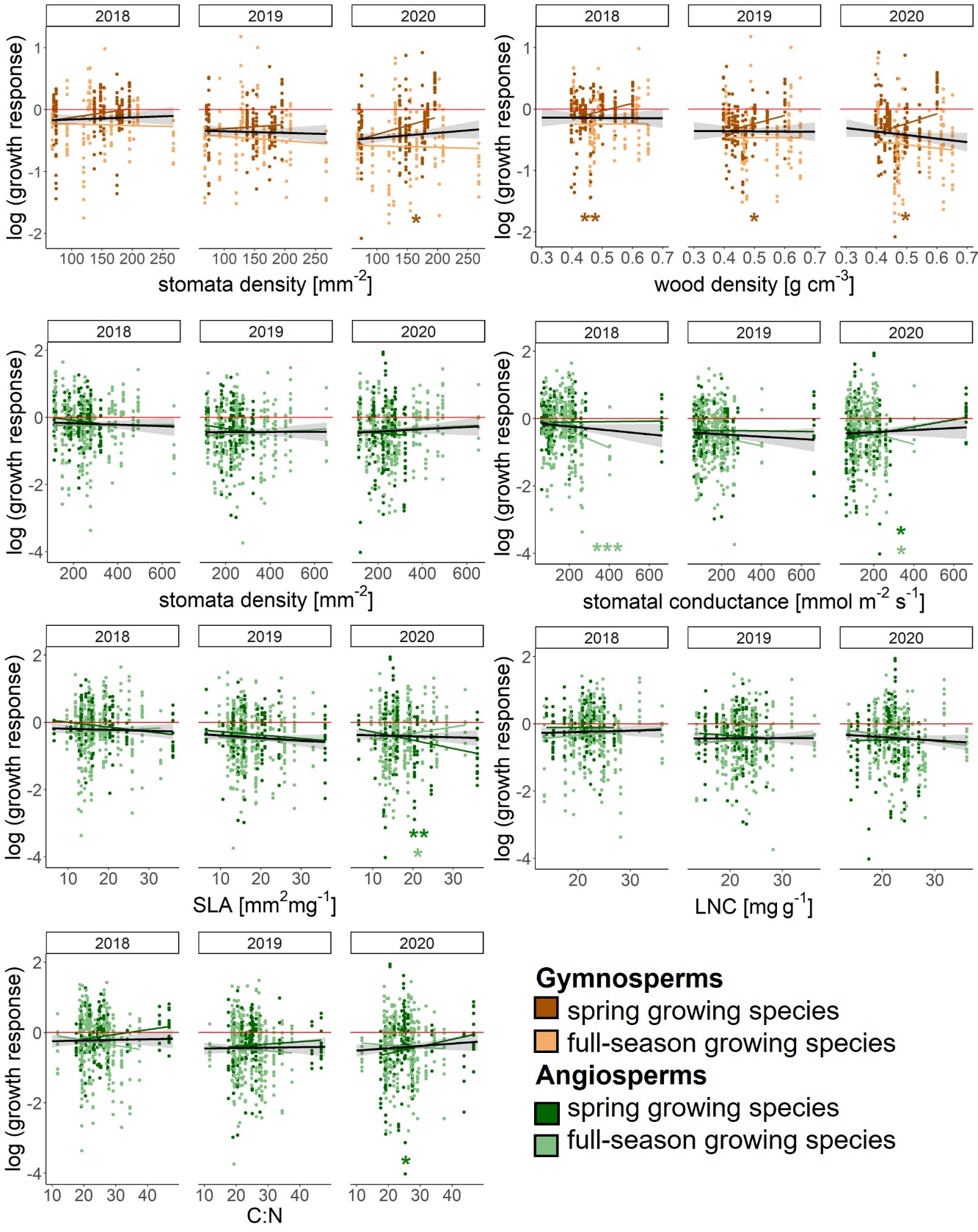


**Figure S12**: Trait-driven responses in growth for gymnosperms and angiosperms during the 2018–2020 drought based on linear mixed-effects model fits. Shown are all not significant relationships. Zero corresponds to a comparable growth in drought and climatically normal years (the mean of the reference years 2016/2017) shown as a red zero-line, while negative growth responses indicate growth reductions. Dark-coloured dots indicate *spring growing*, while light-coloured dots indicate *full-season growing* species. The black lines with 95% confidence intervals show the overall trends. Coloured asterisks below indicate significant relationships for each growth program (* *p*<0.05, ** *p*<0.01, *** *p*<0.001) within the year.

Figure S13


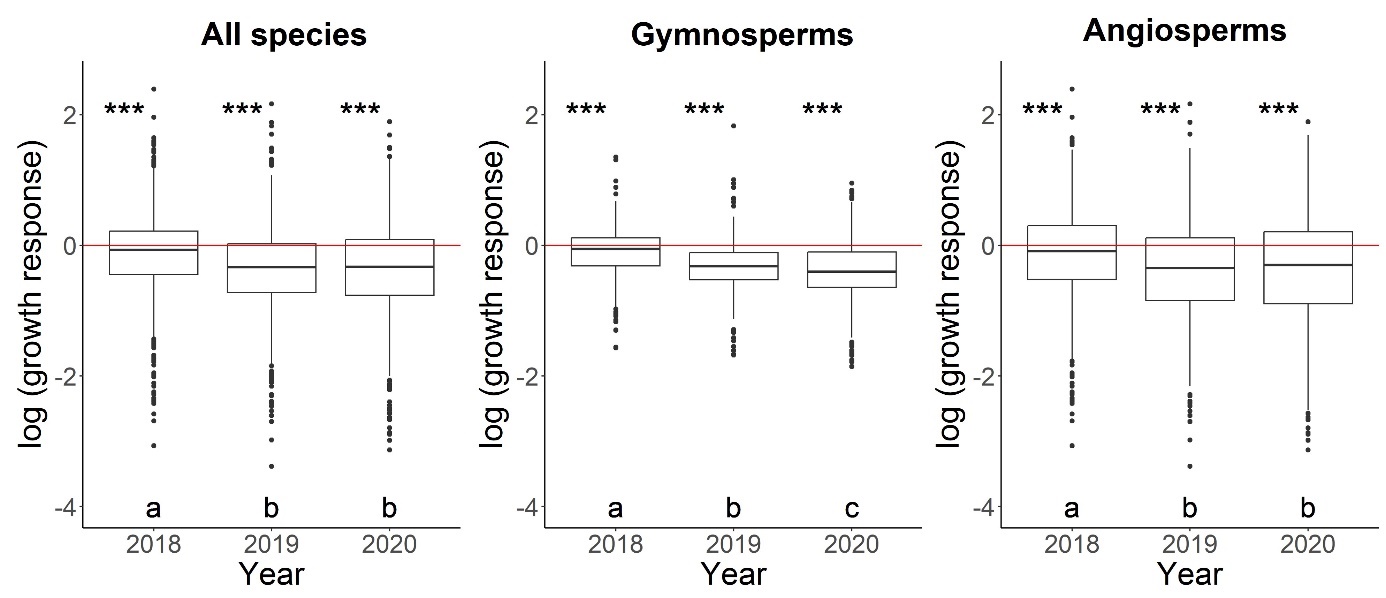


**Figure S13**: Growth response with only year 2017 as reference. Boxplots show the growth response of trees during the drought years 2018–2020 compared to the growth in only one reference year (2017) shown as red zero-line. Across species, the asterisks indicate significant differences in growth response (* *p*<0.05, ** *p*<0.01, *** *p*<0.001), compared to the growth response in the reference year. The significant differences between the years were tested with a post-hoc test and are indicated by the characters (a, b, c). Similarly, significant (*p*<0.05) reductions in growth response were found when analysing the gymnosperms and the angiosperms separated.

Figure S14


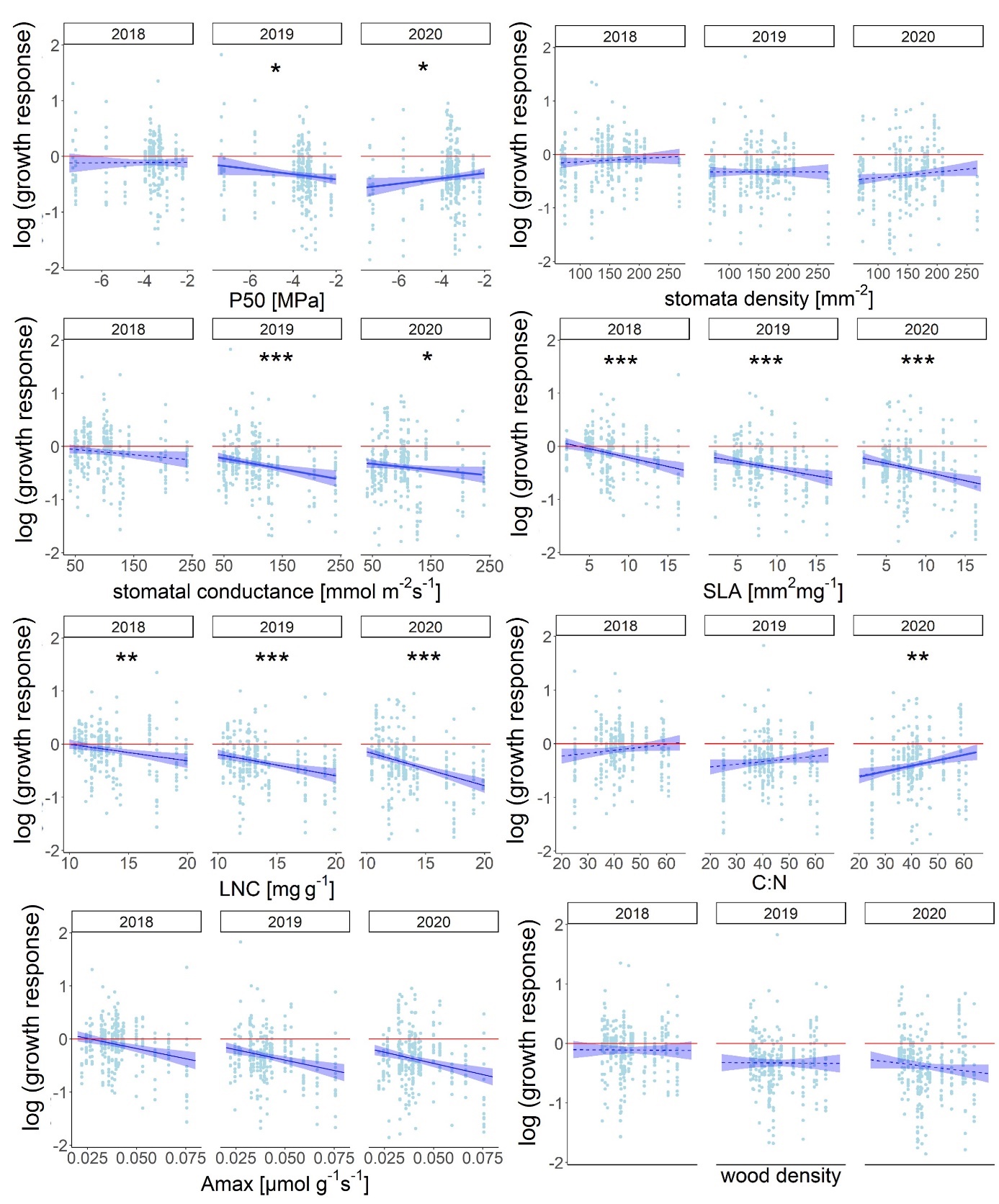


**Figure S14**: Trait-driven responses in growth response for gymnosperms with only 2017 as reference year. Shown are relationships between functional traits and the growth response of gymnosperm trees during the drought years 2018–2020 based on linear mixed-effects model fits. Growth response is depicted compared to tree growth in the reference year 2017 shown as red zero-line. The asterisks indicate significant relationships (* *p*<0.05, ** *p*<0.01, *** *p*<0.001), while a dashed line symbolizes a non-significant relationship. Shaded bands show the 95% confidence intervals.

Figure S15


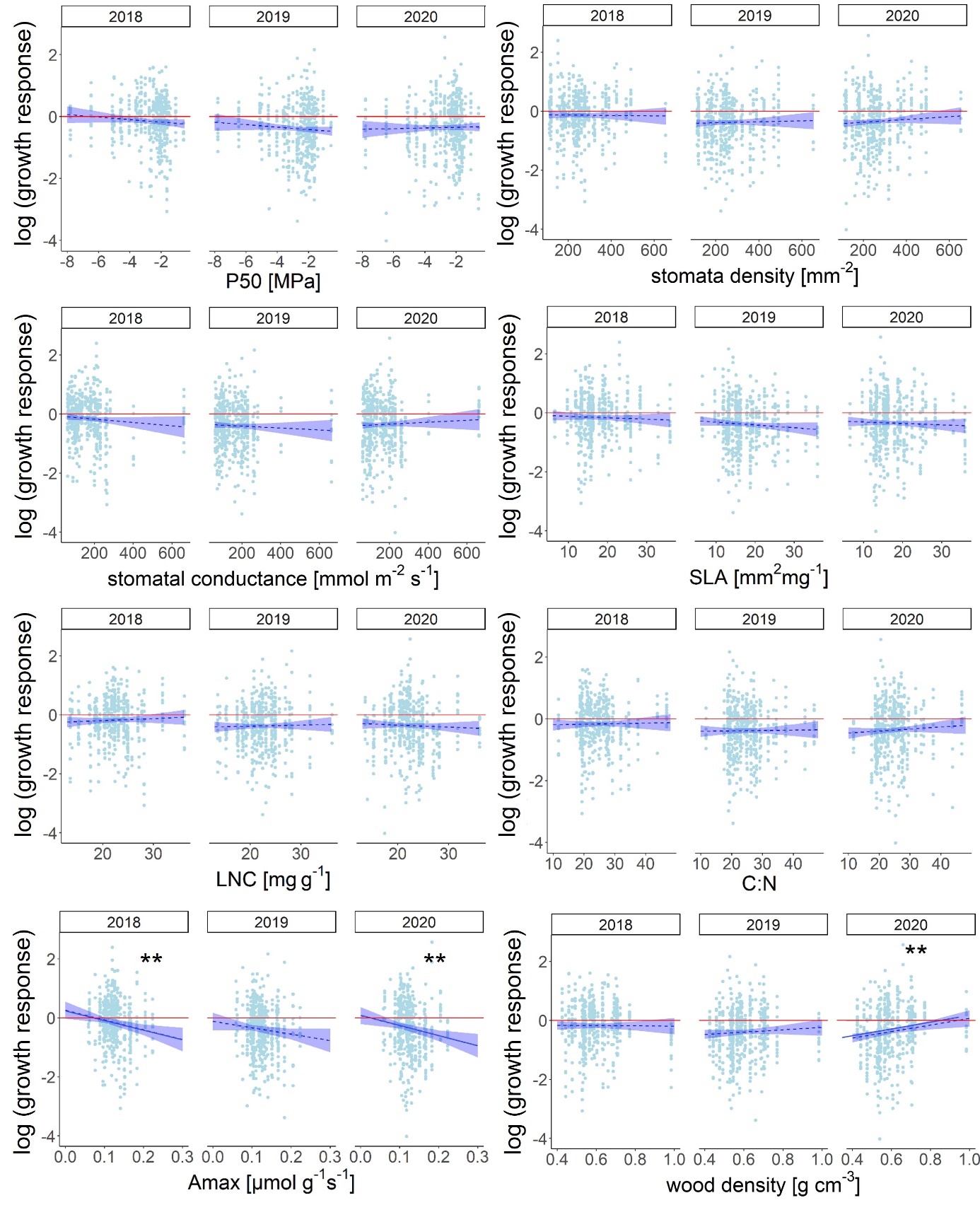


**Figure S15**: Trait-driven growth responses for angiosperms with only 2017 as reference year. Shown are relationships between functional traits and the growth response of angiosperm trees during the drought years 2018–2020 based on linear mixed-effects model fits. Growth response is depicted compared to tree growth in the reference year 2017 shown as red zero-line. The asterisks indicate significant relationships (* *p*<0.05, ** *p*<0.01, *** *p*<0.001), while a dashed line symbolizes a non-significant relationship. Shaded bands show the 95% confidence intervals.

**Table S16**: Species list of all investigated species and their attribution to the response types. Species names are coloured into orange gymnosperms and green angiosperms. The response types result from the growth response pattern according to the decision tree in Figure S9. The green colours for growth response indicate not significant (0) and significant positive (darker green, 0+*) growth response in the years 2018, 2019 and 2020, while the red colour indicate significant negative growth response. The asterisks indicate the levels of significance (* *p*<0.05, ** *p*<0.01, *** *p*<0.001). R^2^m and R^2^c are the predictive power of the single species model, without and with the random effect included, respectively. The branches are the numbers of the individual branches included in the analysis.

| **Species** | **Growth program** | **Response type** | **Growth response 2018** | **Growth response 2019** | **Growth response 2020** | **R^2^m** | **R^2^c** | **Branches** |
| --- | --- | --- | --- | --- | --- | --- | --- | --- |
| *Abies alba* Mill. | spring growing | Late sufferer | 0 | * | *** | 0.37 | 0.48 | 15 |
| *Abies balsamea* (L.) Mill. | spring growing | Sufferer | *** | *** | *** | 0.44 | 0.59 | 12 |
| *Abies grandis* (Douglas ex D. Don) Lindley | spring growing | Resister | 0 | 0 | 0 | 0.03 | 0.49 | 15 |
| *Acer campestre* L. | full-season growing | Late sufferer | 0 | 0 | * | 0.19 | 0.44 | 9 |
| *Acer negundo* L. | spring growing | Late sufferer | 0 | * | *** | 0.31 | 0.45 | 12 |
| *Acer platanoides* L. | full-season growing | Recoverer | * | *** | 0 | 0.33 | 0.50 | 15 |
| *Acer pseudoplatanus* L. | spring growing | Resister | 0 | 0 | 0 | 0.02 | 0.41 | 15 |
| *Acer saccharinum* L. | full-season growing | Sufferer | *** | *** | ** | 0.32 | 0.42 | 15 |
| *Aesculus hippocastanum* L. | spring growing | Recoverer | 0 | * | 0 | 0.17 | 0.38 | 12 |
| *Carpinus betulus* L. | full-season growing | Recoverer | 0 | * | 0 | 0.13 | 0.25 | 15 |
| *Castanea sativa* Mill. | full-season growing | Resister | 0 | 0 | 0 | 0.03 | 0.03 | 11 |
| *Cedrus deodara* (Roxb. ex D.Don) G.Don | full-season growing | Sufferer | *** | ** | ** | 0.37 | 0.41 | 6 |
| *Cedrus libani* A. Rich. | full-season growing | Late sufferer | 0 | 0 | ** | 0.36 | 0.36 | 9 |
| *Celtis occidentalis* L. | full-season growing | Recoverer | * | 0 | 0 | 0.25 | 0.42 | 6 |
| *Corylus avellana* L. | spring growing | Resister | 0 | 0 | 0 | 0.08 | 0.20 | 12 |
| *Corylus colurna* L. | full-season growing | Recoverer | 0 | *** | 0 | 0.33 | 0.62 | 9 |
| *Crataegus laevigata* (Poir.) DC. | spring growing | Resister | 0 | 0 | 0 | 0.06 | 0.32 | 12 |
| *Crataegus monogyna* Jacq. | spring growing | Late sufferer | 0 | 0 | * | 0.11 | 0.22 | 15 |
| *Euonymus europaeus* L. | spring growing | Late sufferer | 0 | ** | * | 0.21 | 0.46 | 9 |
| *Fagus sylvatica* L. | full-season growing | Recoverer | 0 | * | 0 | 0.09 | 0.27 | 15 |
| *Fraxinus excelsior* L. | spring growing | Recoverer | 0 | * | 0 | 0.08 | 0.52 | 9 |
| *Ilex aquifolium* L. | spring growing | Resister | 0 | 0 | 0 | 0.09 | 0.09 | 6 |
| *Juglans nigra* L. | full-season growing | Sufferer | *** | *** | ** | 0.37 | 0.37 | 14 |
| *Juglans regia* L. | full-season growing | Sufferer | ** | *** | ** | 0.41 | 0.60 | 6 |
| *Juniperus communis* L. | full-season growing | Late sufferer | 0 | 0 | ** | 0.13 | 0.39 | 12 |
| *Larix decidua* Mill. | full-season growing | Sufferer | ** | *** | *** | 0.54 | 0.65 | 15 |
| *Larix kaempferi* (Lamb.) Carr. | full-season growing | Sufferer | *** | *** | *** | 0.50 | 0.69 | 15 |
| *Malus sylvestris* (L.) Mill | full-season growing | Late sufferer | 0 | 0 | ** | 0.16 | 0.50 | 9 |
| *Mespilus germanica* L. | full-season growing | Recoverer | 0 | * | 0 | 0.33 | 0.58 | 6 |
| *Ostrya carpinifolia* Scop. | full-season growing | Late sufferer | 0 | 0 | * | 0.24 | 0.34 | 6 |
| *Picea abies* (L.) H. Karst. | spring growing | Late sufferer | 0 | ** | ** | 0.22 | 0.22 | 15 |
| *Picea glauca* (Moench) Voss | spring growing | Recoverer | 0 | *** | 0 | 0.46 | 0.52 | 15 |
| *Picea pungens* Engelm. | spring growing | Late sufferer | 0 | *** | *** | 0.38 | 0.49 | 15 |
| *Picea sitchensis* (Bong.) Carr. | full-season growing | Resister | 0+** | 0 | 0 | 0.14 | 0.57 | 15 |
| *Pinus cembra* L. | spring growing | Late sufferer | 0 | *** | *** | 0.48 | 0.58 | 15 |
| *Pinus contorta* Douglas | spring growing | Late sufferer | 0 | ** | * | 0.09 | 0.54 | 15 |
| *Pinus mugo* Turra | spring growing | Resister | 0 | 0 | 0+*** | 0.28 | 0.33 | 15 |
| *Pinus nigra* J.F. Arnold | full-season growing | Late sufferer | 0 | *** | *** | 0.49 | 0.70 | 15 |
| *Pinus ponderosa* Douglas ex C. Lawson | spring growing | Sufferer | * | *** | *** | 0.47 | 0.53 | 15 |
| *Pinus strobus* L. | spring growing | Late sufferer | 0 | * | * | 0.10 | 0.45 | 12 |
| *Pinus sylvestris* L. | spring growing | Late sufferer | 0 | *** | *** | 0.44 | 0.44 | 15 |
| *Platanus acerifolia* (Aiton) Willd. | full-season growing | Sufferer | ** | *** | *** | 0.38 | 0.47 | 12 |
| *Populus nigra* L. | full-season growing | Sufferer | *** | *** | *** | 0.48 | 0.50 | 12 |
| *Populus tremula* L. | full-season growing | Sufferer | ** | * | *** | 0.20 | 0.25 | 15 |
| *Prunus avium* L. | spring growing | Late sufferer | 0 | 0 | ** | 0.15 | 0.36 | 15 |
| *Prunus mahaleb* L. | full-season growing | Resister | 0 | 0 | 0 | 0.07 | 0.12 | 15 |
| *Prunus padus* L. | spring growing | Recoverer | 0 | * | 0 | 0.15 | 0.41 | 15 |
| *Prunus serotina* Ehrh. | spring growing | Late sufferer | 0 | ** | *** | 0.35 | 0.35 | 15 |
| *Pseudotsuga menziesii* (Mirbel) Franco | full-season growing | Sufferer | *** | *** | *** | 0.63 | 0.78 | 15 |
| *Pyrus communis* L. | spring growing | Late sufferer | 0 | 0 | ** | 0.35 | 0.35 | 6 |
| *Quercus cerris* L. | full-season growing | Resister | 0+* | 0 | 0 | 0.06 | 0.17 | 15 |
| *Quercus pubescens* Willd. | full-season growing | Resister | 0 | 0 | 0 | 0.09 | 0.17 | 9 |
| *Quercus robur* L. | full-season growing | Resister | 0 | 0 | 0 | 0.06 | 0.10 | 9 |
| *Quercus rubra* L. | full-season growing | Recoverer | 0+* | ** | 0 | 0.32 | 0.51 | 15 |
| *Robinia pseudoacacia* L. | full-season growing | Sufferer | * | *** | *** | 0.27 | 0.55 | 9 |
| *Salix alba* L. | full-season growing | Sufferer | *** | ** | *** | 0.32 | 0.32 | 12 |
| *Salix caprea* L. | full-season growing | Sufferer | ** | * | ** | 0.32 | 0.32 | 6 |
| *Salix cinerea* L. | spring growing | Late sufferer | 0 | * | * | 0.28 | 0.31 | 6 |
| *Salix pentandra* L. | full-season growing | Sufferer | * | *** | *** | 0.77 | 0.81 | 9 |
| *Sorbus domestica* L. | full-season growing | Resister | 0 | 0 | 0 | 0.14 | 0.38 | 12 |
| *Sorbus intermedia* (Ehrh.) Pers. | full-season growing | Recoverer | * | * | 0 | 0.15 | 0.26 | 12 |
| *Sorbus torminalis* (L.) Crantz | full-season growing | Recoverer | 0 | *** | 0 | 0.30 | 0.55 | 12 |
| *Taxus baccata* L. | full-season growing | Sufferer | ** | *** | *** | 0.32 | 0.41 | 15 |
| *Tilia cordata* Mill. | full-season growing | Recoverer | 0 | * | 0 | 0.17 | 0.31 | 15 |
| *Tilia platyphyllos* Scop. | full-season growing | Resister | 0+** | 0 | 0 | 0.11 | 0.53 | 12 |
| *Tilia tomentosa* Moench | spring growing | Late sufferer | 0 | * | * | 0.14 | 0.33 | 15 |
| *Tsuga canadensis* (L.) Carrière | full-season growing | Sufferer | *** | *** | *** | 0.69 | 0.73 | 9 |
| *Tsuga heterophylla* (Raf.) Sarg. | full-season growing | Recoverer | 0 | * | 0 | 0.16 | 0.54 | 6 |
| *Ulmus glabra* Huds. | spring growing | Late sufferer | 0 | 0 | ** | 0.27 | 0.55 | 6 |
| *Ulmus laevis* Pall. | spring growing | Sufferer | ** | ** | *** | 0.26 | 0.34 | 15 |
| *Ulmus minor* Mill. | full-season growing | Late sufferer | 0 | 0 | ** | 0.14 | 0.40 | 12 |

**Table S17**: Growth response explained by single traits based on linear mixed-effects model fits. The asterisks indicate significant relationships (* *p*<0.05, ** *p*<0.01, *** *p*<0.001), while the colours indicate the direction of the relationship (green=positive, red=negative).

| **Gymnosperms** | | | | | | | | | | | | | | | | | | | |
| --- | --- | --- | --- | --- | --- | --- | --- | --- | --- | --- | --- | --- | --- | --- | --- | --- | --- | --- | --- |
|  | **2018** | | | | **2019** | | | | **2020** | | | | **interaction with growth program** | **2018** | | **2019** | | **2020** | |
|  | ***spring-growing*** | **sig.** | ***full-season growing*** | **sig.** | ***spring growing*** | **sig.** | ***full-season growing*** | **sig.** | ***spring growing*** | **sig.** | ***full-season growing*** | **sig.** |  | **all** | **sig.** | **all** | **sig.** | **all** | **sig.** |
| **P50** | -0.175 | * | -0.032 |  | -0.196 | ** | -0.094 | *** | -0.243 | *** | 0.005 |  |  | -0.010 |  | -0.057 | * | 0.034 |  |
| **Stomata density** | 0.001 |  | <-0.001 |  | 0.001 |  | -0.001 |  | 0.003 | *** | <-0.001 |  |  | <0.001 |  | <-0.001 |  | 0.001 |  |
| **Stomatal conductance** | 0.001 |  | -0.001 |  | <0.001 |  | -0.002 | *** | 0.003 |  | -0.001 |  |  | -0.001 | * | -0.002 | *** | -0.001 | * |
| **SLA** | -0.022 | * | -0.051 | *** | -0.013 |  | -0.044 | *** | -0.011 |  | -0.055 | *** | * | -0.040 | *** | -0.033 | *** | -0.039 | *** |
| **LNC** | -0.025 |  | -0.030 | * | -0.062 | ** | -0.028 | * | -0.131 | *** | -0.035 | ** |  | -0.037 | *** | -0.045 | *** | -0.069 | *** |
| **C:N** | 0.009 |  | 0.006 |  | 0.015 | ** | 0.003 |  | 0.030 | *** | 0.004 |  | * | 0.006 | * | 0.006 |  | 0.011 | *** |
| **Amax** | -8.160 | * | -8.150 | ** | -4.660 |  | -10.280 | *** | -7.300 | * | -8.030 | ** |  | -9.230 | *** | -9.420 | *** | -10.000 | *** |
| **Wood density** | 1.222 | * | 0.038 |  | 1.356 | * | -0.005 |  | 1.582 | ** | -0.563 |  |  | -0.030 |  | -0.030 |  | -0.572 |  |
| **PC1** | 0.115 | ** | 0.051 | ** | 0.072 |  | 0.048 |  | 0.099 | * | 0.055 | ** |  | 0.066 | *** | 0.059 | *** | 0.071 | *** |
| **PC2** | -0.178 | ** | -0.013 |  | -0.148 |  | -0.018 |  | -0.063 |  | 0.006 |  |  | <0.001 |  | 0.008 |  | 0.041 |  |
| **Angiosperms** | | | | | | | | | | | | | | | | | | | |
|  | **2018** | | | | **2019** | | | | **2020** | | | | **interaction with growth program** | **2018** | | **2019** | | **2020** | |
|  | ***spring-growing*** | **sig.** | ***full-season growing*** | **sig.** | ***spring growing*** | **sig.** | ***full-season growing*** | **sig.** | ***spring growing*** | **sig.** | ***full-season growing*** | **sig.** |  | **all** | **sig.** | **all** | **sig.** | **all** | **sig.** |
| **P50** | -0.020 |  | -0.113 | * | -0.033 |  | -0.093 | * | 0.019 |  | -0.109 | * |  | -0.066 | ** | -0.064 | * | -0.015 |  |
| **Stomata density** | <-0.001 |  | <0.001 |  | -0.001 |  | <0.001 |  | <-0.001 |  | <0.001 |  |  | <-0.001 |  | <0.001 |  | <0.001 |  |
| **Stomatal conductance** | <0.001 |  | -0.002 | *** | <-0.001 |  | -0.001 |  | 0.001 | * | -0.002 | * | * | <-0.001 |  | <-0.001 |  | <0.001 |  |
| **SLA** | -0.014 |  | 0.006 |  | -0.010 |  | -0.006 |  | -0.025 | ** | 0.019 | * |  | -0.003 |  | -0.008 |  | -0.003 |  |
| **LNC** | <0.001 |  | 0.014 |  | -0.011 |  | 0.010 |  | 0.004 |  | -0.020 |  |  | 0.004 |  | <0.001 |  | -0.010 |  |
| **C:N** | 0.013 |  | -0.015 |  | 0.007 |  | -0.007 |  | 0.020 | * | -0.006 |  |  | 0.002 |  | 0.001 |  | 0.007 |  |
| **Amax** | -1.979 |  | -4.312 | ** | -2.931 |  | -1.554 |  | -0.515 |  | -5.159 | *** |  | -3.148 | ** | -2.242 |  | -2.837 | ** |
| **Wood density** | -0.149 |  | 0.136 |  | 0.176 |  | 0.573 |  | 0.686 |  | 1.056 | ** |  | -0.141 |  | 0.298 |  | 1.015 | ** |
| **PC1** | -0.056 |  | -0.027 |  | -0.064 |  | -0.023 |  | -0.045 |  | -0.062 |  |  | -0.048 |  | -0.047 |  | -0.051 |  |
| **PC2** | 0.004 |  | 0.133 | ** | 0.005 |  | 0.069 |  | -0.024 |  | 0.144 | *** |  | 0.049 |  | 0.019 |  | 0.087 | ** |

**Table S18**: Goodness of fit of the trait models with interaction (trait * year * growth program) and without the growth program interaction (trait * year). The marginal R^2^ (R^2^m) shows the variation explained by fixed and the conditional R^2^ (R^2^c) the variation explained by fixed and random effects. Model results are presented in Figure 5, 6, Figure S12.

| **Gymnosperms** | | | | | |
| --- | --- | --- | --- | --- | --- |
|  |  | **trait * year *  growth program** | | **trait * year** | |
|  | **number of species** | **R^2^m** | **R^2^c** | **R^2^m** | **R^2^c** |
| **P50** | 23 | 0.265 | 0.464 | 0.178 | 0.430 |
| **Stomata density** | 23 | 0.242 | 0.452 | 0.171 | 0.424 |
| **Stomatal conductance** | 23 | 0.247 | 0.456 | 0.198 | 0.435 |
| **SLA** | 20 | 0.323 | 0.498 | 0.271 | 0.476 |
| **LNC** | 21 | 0.306 | 0.503 | 0.252 | 0.476 |
| **C:N** | 22 | 0.273 | 0.468 | 0.186 | 0.429 |
| **Amax** | 23 | 0.272 | 0.462 | 0.235 | 0.444 |
| **Wood density** | 23 | 0.243 | 0.452 | 0.171 | 0.424 |
| **PC1** | 19 | 0.291 | 0.490 | 0.245 | 0.470 |
| **PC2** | 19 | 0.260 | 0.483 | 0.194 | 0.456 |
| **Angiosperms** | | | | | |
|  |  | **trait * year *  growth program** | | **trait * year** | |
|  | **number of species** | **R^2^m** | **R^2^c** | **R^2^m** | **R^2^c** |
| **P50** | 42 | 0.085 | 0.301 | 0.076 | 0.295 |
| **Stomata density** | 44 | 0.074 | 0.292 | 0.067 | 0.284 |
| **Stomatal conductance** | 44 | 0.096 | 0.303 | 0.072 | 0.289 |
| **SLA** | 45 | 0.084 | 0.297 | 0.068 | 0.284 |
| **LNC** | 43 | 0.080 | 0.299 | 0.069 | 0.286 |
| **C:N** | 44 | 0.083 | 0.295 | 0.068 | 0.284 |
| **Amax** | 45 | 0.090 | 0.298 | 0.085 | 0.302 |
| **Wood density** | 45 | 0.085 | 0.302 | 0.078 | 0.296 |
| **PC1** | 39 | 0.080 | 0.298 | 0.073 | 0.289 |
| **PC2** | 39 | 0.092 | 0.302 | 0.073 | 0.290 |

**Table S19**: PC loadings of the PCAs depicting the gymnosperms and angiosperms. Loadings for PC1, PC2, PC3 and PC4 are shown.

| **Gymnosperms** | | | | |
| --- | --- | --- | --- | --- |
|  | **PC1** | **PC2** | **PC3** | **PC4** |
| **Standard deviation** | 1.744 | 1.482 | 1.024 | 0.725 |
| **Proportion of Variance** | 0.380 | 0.275 | 0.131 | 0.066 |
| **Cumulative Proportion** | 0.380 | 0.655 | 0.786 | 0.851 |
|  |  |  |  |  |
|  | **PC1** | **PC2** | **PC3** | **PC4** |
| **P50** | -0.025 | 0.598 | -0.149 | 0.088 |
| **stomata density** | 0.403 | 0.098 | -0.455 | -0.207 |
| **stomatal conductance** | -0.280 | -0.304 | -0.603 | -0.470 |
| **SLA** | -0.439 | 0.186 | -0.356 | 0.241 |
| **LNC** | -0.451 | -0.333 | 0.066 | -0.186 |
| **C:N** | 0.355 | -0.202 | -0.505 | 0.483 |
| **A_max_** | -0.485 | 0.182 | -0.127 | 0.433 |
| **wood density** | 0.032 | -0.566 | 0.071 | 0.464 |
|  |  |  |  |  |
| **Angiosperms** | | | | |
|  | **PC1** | **PC2** | **PC3** | **PC4** |
| **Standard deviation** | 1.589 | 1.251 | 1.152 | 0.973 |
| **Proportion of Variance** | 0.315 | 0.196 | 0.166 | 0.118 |
| **Cumulative Proportion** | 0.315 | 0.511 | 0.677 | 0.795 |
|  |  |  |  |  |
|  | **PC1** | **PC2** | **PC3** | **PC4** |
| **P50** | 0.277 | 0.378 | -0.249 | 0.625 |
| **stomata density** | 0.135 | 0.653 | -0.251 | 0.052 |
| **stomatal conductance** | 0.246 | -0.283 | -0.640 | -0.119 |
| **SLA** | 0.417 | 0.198 | 0.091 | -0.553 |
| **LNC** | 0.509 | 0.080 | 0.270 | -0.004 |
| **C:N** | -0.464 | 0.005 | -0.277 | 0.142 |
| **A_max_** | 0.347 | -0.414 | -0.416 | 0.015 |
| **wood density** | -0.275 | 0.365 | -0.367 | -0.517 |

**Table S20**: Distribution range of traits. Minimum and maximum trait expression of the whole dataset, separated by clade and including the species of its occurrence.

| **Gymnosperms** | | | | |
| --- | --- | --- | --- | --- |
|  | **minimum** |  | **maximum** |  |
| **P50** | - 7.344 | *Cedrus libani* | - 2.228 | *Abies balsamea* |
| **Stomata density** | 68.217 | *Larix decidua* | 267.837 | *Tsuga canadensis* |
| **Stomatal conductance** | 41.312 | *Cedrus deodara* | 241.069 | *Larix decidua* |
| **SLA** | 2.134 | *Picea pungens* | 16.339 | *Larix kaempferi* |
| **LNC** | 10.440 | *Pinus nigra* | 19.875 | *Larix decidua* |
| **C:N** | 24.954 | *Larix kaempferi* | 60.743 | *Tsuga canadensis* |
| **Amax** | 0.022 | *Cedrus deodara* | 0.076 | *Larix kaempferi* |
| **Wood density** | 0.397 | *Abies grandis* | 0.652 | *Taxus baccata* |
|  |  |  |  |  |
| **Angiosperms** | | | | |
|  | **minimum** |  | **maximum** |  |
| **P50** | - 7.798 | *Crataegus laevigata* | - 0.561 | *Ulmus glabra* |
| **Stomata density** | 113.147 | *Crataegus laevigata* | 653.178 | *Celtis occidentalis* |
| **Stomatal conductance** | 62.487 | *Prunus serotina* | 662.397 | *Corylus avellana* |
| **SLA** | 6.508 | *Ilex aquifolium* | 35.957 | *Ulmus laevis* |
| **LNC** | 13.570 | *Sorbus intermedia* | 36.126 | *Robinia pseudoacacia* |
| **C:N** | 11.715 | *Robinia pseudoacacia* | 46.773 | *Aesculus hippocastanum* |
| **Amax** | 0.059 | *Ilex aquifolium* | 0.223 | *Robinia pseudoacacia* |
| **Wood density** | 0.425 | *Prunus padus* | 0.984 | *Acer saccharinum* |
